# Silver nanoparticles selectively treat triple‐negative breast cancer cells without affecting non‐malignant breast epithelial cells in vitro and in vivo

**DOI:** 10.1096/fba.2019-00021

**Published:** 2019-09-30

**Authors:** Jessica Swanner, Cale D. Fahrenholtz, Iliana Tenvooren, Brian W. Bernish, James J. Sears, Allison Hooker, Cristina M. Furdui, Elizabeth Alli, Wencheng Li, George L. Donati, Katherine L. Cook, Pierre‐Alexandre Vidi, Ravi Singh

**Affiliations:** ^1^ Department of Cancer Biology Wake Forest School of Medicine Winston Salem NC USA; ^2^ Salem College Winston Salem NC USA; ^3^ Department of Internal Medicine Section on Molecular Medicine Wake Forest School of Medicine Winston‐Salem NC USA; ^4^ Comprehensive Cancer Center of Wake Forest Baptist Medical Center Winston‐Salem NC USA; ^5^ Department of Pathology Wake Forest School of Medicine Winston‐Salem NC USA; ^6^ Department of Chemistry Wake Forest University Winston‐Salem NC USA; ^7^ Department of Surgery Wake Forest School of Medicine Winston‐Salem NC USA

**Keywords:** DNA damage, nanomedicine, redox, toxicity, unfolded protein response

## Abstract

Silver nanoparticles (AgNPs) show promise for treatment of aggressive cancers including triple‐negative breast cancer (TNBC) in preclinical cancer models. For clinical development of AgNP‐based therapeutics, it will be necessary to clearly define the specific physicochemical features of the nanoparticles that will be used, and to tie these properties to biological outcomes. To fill this knowledge gap, we performed thorough structure/function, mechanistic, safety, and efficacy studies to assess the potential for AgNPs to treat TNBC. We establish that AgNPs, regardless of size, shape, or stabilizing agent, are highly cytotoxic to TNBC cells at doses that are not cytotoxic to non‐malignant breast epithelial cells. In contrast, TNBC cells and non‐malignant breast epithelial cells are similarly sensitive to exposure to silver cation (Ag^+^), indicating that the nanoparticle formulation is essential for the TNBC‐specific cytotoxicity. Mechanistically, AgNPs are internalized by both TNBC and non‐malignant breast cells, but are rapidly degraded only in TNBC cells. Exposure to AgNPs depletes cellular antioxidants and causes endoplasmic reticulum stress in TNBC cells without causing similar damage in non‐malignant breast epithelial cells. AgNPs also cause extensive DNA damage in 3D TNBC tumor nodules in vitro, but do not disrupt the normal architecture of breast acini in 3D cell culture, nor cause DNA damage or induce apoptosis in these structures. Lastly, we show that systemically administered AgNPs are effective at non‐toxic doses for reducing the growth of TNBC tumor xenografts in mice. This work provides a rationale for development of AgNPs as a safe and specific TNBC treatment.

**Abbreviations**
3D: three dimensional
Ag^+^: silver cation
Ag^0^: silver metal
AgNP: silver nanoparticle
AnnV: annexin V
APC: allophycocyanin
AuNP: gold nanoparticle
BCA: bicinchoninic acid
CHOP: CCAAT‐enhancer‐binding protein homologous protein
Cyt D: cytochalasin D
DAPI: 4′,6‐diamidino‐2‐phenylindole
DLS: dynamic light scattering
eIF2α: alpha subunit of the eukaryotic translation initiation factor 2 complex
ER: endoplasmic reticulum
FBS: fetal bovine serum
GRP78: glucose regulated protein 78
GSH: glutathione
GSSG: glutathione dimer
HER2: human epidermal growth factor receptor 2
HRP: horse radish peroxidase
IC50: half maximal inhibitory concentration
ICP‐MS: inductively coupled plasma mass spectrometry
MTT: 3‐(4,5‐dimethylthiazol‐2‐yl)‐2,5‐diphenyltetrazolium bromide
NADPH: nicotinamide adenine dinucleotide phosphate
NTA: nanoparticle tracking analysis
O_2_^−^: superoxide radical
PBS: Phosphate buffered saline
PERK: protein kinase RNA‐like endoplasmic reticulum kinase
PI: propidium iodide
PVDF: polyvinylidene fluoride
PVP: polyvinylpyrrolidone
ROS: reactive oxygen species
TNBC: triple‐negative breast cancer
UPR: unfolded protein response

## 1 INTRODUCTION

Triple‐negative breast cancer (TNBC) is an aggressive, malignant neoplasia characterized by lack or decreased expression of estrogen, progesterone, and human epidermal growth factor receptors. As a consequence, TNBC patients do not benefit from modern receptor‐targeted therapies.^1^ Moreover, TNBC patients have a significantly higher risk of recurrence and death than patients with other types of breast cancer.^2^ Disease heterogeneity has limited the development of molecularly targeted therapeutics.^3^ The current standard of care for TNBC involves surgical resection of the primary tumor preceded or followed by ionizing radiation and a cocktail of chemotherapies.^4^ Invasive surgeries require long recovery periods and ionizing radiation may have severe off‐targets effects, such as the development of ischemic heart disease due to the irradiation of nearby healthy cardiac tissue.^5^ Standard chemotherapeutics, including taxanes, possess dose‐limiting toxicities due to significant off‐target side effects, elevated risk for secondary, therapy‐related cancers,^6^ and offer little selectivity for the TNBC cells.^7^

Many studies have investigated nanoparticles as drug carriers with the potential to increase bioavailability and delivery of small molecules to tumors, but unfortunately, the majority of nanoparticles fail to find their tumor target,^8^ which raises concerns about their off‐target toxicity. Several studies showed that metallic, drug‐free, nanoparticles, made of materials including gold, silver, iron, or gadolinium, exhibit unique cytotoxicity profiles that enable them to exploit specific vulnerabilities in cancer cells without causing significant off‐target toxicity.^9^, ^10^, ^11^, ^12^, ^13^ Among metallic nanoparticles, silver nanoparticles (AgNPs) have high biomedical relevance primarily due to their antimicrobial properties.^14^, ^15^, ^16^ AgNPs are also reported to be cytotoxic to cancers including those of the breast,^17^ ovary,^18^ brain,^19^, ^20^, ^21^ cervix,^22^ liver,^23^ colon,^24^ lung,^25^ pancreas,^26^ and blood.^27^, ^28^, ^29^, ^30^ We recently observed that exposure to AgNPs in vitro was lethal to three TNBC cell lines at doses that were not cytotoxic to non‐cancerous breast cells or immortalized cells derived from kidney, liver, or monocyte/macrophages.^11^ Furthermore, we found that intratumoral injection of AgNPs increased the efficacy of ionizing radiation for treatment of TNBC xenografts in mice.^11^ Clinical data of AgNP usage in human cancer patients are limited. However, a recent case report describes the complete regression of metastatic head and neck cancer in a patient who ingested AgNPs, in the absence of other anticancer therapy, after failure of platinum‐ and taxane‐based chemotherapy, radiation, and surgical resection.^31^

Although these studies support the potential for wider clinical use of AgNPs for cancer therapy, AgNPs are pleotropic stressors, and it is necessary to consider sub‐lethal, off‐target toxicity which could affect their safety and potential for clinical translation.^32^, ^33^ In addition to DNA damage,^11^, ^18^ emerging evidence suggests that AgNPs can cause endoplasmic reticulum (ER) stress which initiates the unfolded protein response (UPR).^34^, ^35^, ^36^ The UPR is an important cellular self‐protection mechanism, but chronic activation of the UPR due to stress that exceeds the capacity for self‐protection leads to apoptosis and cell death.^37^ ER stress is emerging as an Achilles heel for some cancers and exploiting this vulnerability may offer a route to selective cancer therapy.^37^, ^38^, ^39^

Small changes in nanoparticle characteristics may dramatically change their toxicity profile.^33^ Therefore, it is necessary to establish clear structural and physicochemical characterizations and biological function relationships for AgNPs. Monolayer cell cultures offer significant value for screening and mechanistic studies of nanoparticle toxicity, but cell monolayers may not adequately represent the functions of tissues, which have extensive cell‐cell and cell‐matrix interactions.^40^ Such interactions can have a dramatic effect on the sensitivity of the cells to therapy.^41^ Additionally, monolayer cell culture fails to reproduce the barrier aspects of extracellular matrix, which may dramatically influence the diffusion/transport of nanoparticles and affect the exposure of cells.^41^ Hence, testing cytotoxicity in monolayer may not reflect the entire profile of nanoparticle toxicity. Tissue engineering can re‐create the three‐dimensional (3D) geometry, chemistry, function and signaling microenvironment of tissues or tumors.^42^ Evaluation of nanotherapeutics in 3D tissue culture may more accurately recapitulate the complexity of tissues in vivo.^41^, ^42^

In this study, we evaluate the hypothesis that an exploitable vulnerability to AgNPs exists in TNBC cells, and we determine how size, shape, or coating affects the sensitivity of TNBC cells to AgNPs. To determine potential off‐target, sub‐lethal effects of AgNPs, we quantify the impact of AgNPs on ER stress, DNA damage, cell polarity, and apoptosis in non‐cancerous breast epithelial cells using monolayer and 3D tumor organoid cultures in vitro. Furthermore, we perform in vivo experiments to determine the efficacy and tolerability of intravenously administered AgNPs for treatment of TNBC xenografts in mice. These studies help to link cell culture to murine and eventual human studies, and will guide future advancements in the use of AgNPs for treatment of TNBC.

## 2 MATERIAL AND METHODS

### 2.1 Silver nanoparticles

5, 25, 50, and 75 nm in diameter spherical AgNPs stabilized with polyvinylpyrrolidone (PVP; 89.3, 85, 76.5, 74% by mass, respectively), PVP and silica shelled triangular silver nanoplates, and PVP‐stabilized 15 nm gold nanoparticles (AuNPs; 91.6% PVP by mass) were purchased as dried nanopowders from nanoComposix, Inc. Free (ionic) silver represented less than 1% of the total nanoparticle silver content according the manufacturer's specifications. Chitosan‐coated AgNPs were synthesized according to previously published methods.^43^ Nanoparticles were dispersed at a concentration of 20 mg/mL (total nanoparticle weight) in phosphate buffered saline (PBS) (Invitrogen) by bath sonication, and then were diluted in cell culture medium to the final concentration listed in the figures prior to addition to the wells containing cells.

### 2.2 Cell culture

MCF‐7, MCF‐10A, MDA‐MB‐231, MDA‐MB‐468, HCC70, and SUM‐159 were authenticated by, and purchased from the ATCC (Manassas, VA, USA). iMEC cells were provided by Dr Elizabeth Alli (Wake Forest School of Medicine). Non‐neoplastic HMT‐3522 S1 (S1) mammary epithelial cells and their neoplastic derivative HMT‐3522 T4‐2 (T4‐2)^44^ were provided by Dr Mina Bissell (Lawrence Berkeley Laboratory). Cells were grown in complete media as described in Table 1, and were maintained in culture for no longer than 4 months before new cultures were established from low‐passage frozen stocks. S1 cells were used between passages 54 and 60.

**Table 1:** Description of cell culture media used to grow various cell lines described in this work

Cell line	Media formulations
HCC70	RPMI supplemented with penicillin (250 units/mL), streptomycin (250 μg/mL), and 10% fetal bovine serum
HMT‐3522 S1	DMEM/F12 supplemented with 5 μg/mL prolactin, 250 ng/mL insulin, 1.4 μmol/L hydrocortisone, 0.1 nmol/L β‐estradiol, 2.6 ng/mL sodium selenite, 10 μg/mL transferrin, 5 ng/mL epidermal growth factor
HMT‐3522 T4‐2	DMEM/F12 supplemented with 5 μg/mL prolactin, 250 ng/mL insulin, 1.4 μmol/L hydrocortisone, 0.1 nmol/L β‐estradiol, 2.6 ng/mL sodium selenite, 10 μg/mL transferrin
iMEC	DMEM/F12 supplemented with 10 µg/mL insulin, 20 ng/mL hEGF, and 0.5 μg/mL hydrocortisone, and 10% fetal bovine serum
MCF‐10A	DMEM/F12 supplemented with penicillin (250 units/mL), streptomycin (250 μg/mL), 2 mmol/L L‐glutamine, 10 μg/mL insulin, 20 ng/mL epidermal growth factor, 0.5 μg/mL hydrocortisone, and 100 ng/mL Cholera toxin, and 5% heat‐inactivated horse serum
MCF‐7	DMEM/F12 supplemented with penicillin (250 units/mL), streptomycin (250 μg/mL), 2mM L‐glutamine, 10 μg/mL insulin, 10 ng/mL epidermal growth factor, 0.5 μg/mL hydrocortisone, and 10% fetal bovine serum
MDA‐MB‐231	DMEM/F12 supplemented with penicillin (250 units/mL), streptomycin (250 μg/mL), 2 mmol/L L‐glutamine, and 10% fetal bovine serum
MDA‐MB‐468	DMEM supplemented with penicillin (250 units/mL), streptomycin (250 μg/mL), 2 mmol/L L‐glutamine, and 10% fetal bovine serum
SUM‐159	HAM’s F12 supplemented with penicillin (250 units/mL), streptomycin (250 μg/mL), 2mM L‐glutamine, 5 μg/mL insulin, 1 μg/mL hydrocortisone, 10 μmol/L HEPES, and 5% fetal bovine serum

All cell lines were verified to be free from mycoplasma contamination by testing using the MycoAlert Mycoplasma Detection Kit (Lonza). Cells were passaged and medium was changed twice weekly. Cell monolayers were grown on tissue culture‐treated plastics purchased from Corning Life Sciences or on glass coverslips (Thermo Fisher Scientific). Alternatively, S1, T4‐2 and MDA‐MB‐231 cells were cultured in 4‐well chamber slides (EMD Millipore) in the presence of reconstituted basement membrane (Matrigel^®^, Corning) to recapitulate the formation of polarized glandular structures (acini) and tumor nodules, respectively, as previously described.^44^

### 2.3 Dynamic light scattering

Measurements were made using the Zetasizer Nano ZS90 (Malvern Instruments). Size measurements were taken at 25°C using automatic settings and adjusting for the refractive index and viscosity of the solutions in which the nanoparticles were suspended. ζ‐potential was measured in water using disposable folded capillary cells (Malvern Instruments). For ζ‐potential measurements, 50 μL of AgNP stock in PBS, which acted as a source of ions needed to form an electric double layer, was diluted in 1 mL in water.

### 2.4 Nanoparticle tracking analysis

Measurements were made using the Nanosight NS500 (Malvern Instruments) at 25°C. AgNP dispersions (20 mg/mL) were diluted 1:50 000 in degassed, type I (18 MΩ cm, Milli‐Q^®^ (EMD Millipore)) water. The following settings were used for five measurements of each preparation: nanoparticle tracking analysis (NTA) software version 3.1; camera shutter: 32 ms; duration: 90; threshold: 4.

### 2.5 MTT assay

Cells were seeded on 96‐well plates at a density of 3000‐6000 cells per well (depending upon cell line) in 200 μL of complete media, recovered for 18 hours, and were then exposed to AgNPs or doxorubicin. To inhibit the uptake of AgNPs, MDA‐MB‐231 cells were treated with AgNPs in the presence of 1 µmol/L Cytochalasin D (Cyt D) (Sigma‐Aldrich) for 6 hours. Treatment media was removed, and fresh growth media was added for an additional 42 hours. At the appropriate time point, media containing AgNPs or doxorubicin were replaced with 200 µL of media containing 0.5 mg/mL 3‐(4,5‐dimethylthiazol‐2‐yl)‐2,5‐diphenyltetrazolium bromide (MTT; Sigma‐Aldrich) and incubated for 1 hours at 37°C. Medium was removed, and cells were lysed in 200 μL of DMSO and read using a Molecular Devices (San Jose, CA, USA) Emax Precision Microplate Reader at 560 nm and corrected for background at 650 nm. To control for possible interference of AgNPs with absorbance readings, a set of control wells, in which cells were treated with AgNPs then solubilized using dimethylsulfoxide (DMSO; Thermo Fisher Scientific) without being exposed to MTT, was included. For all AgNP doses (0‐500 µg/mL), absorbance readings were at background level, equivalent to empty wells, indicating no interference with the assay.

### 2.6 Inductively coupled plasma mass spectrometry

MDA‐MB‐231 and MCF‐10A cells were grown in 60 mm tissue culture plates. Cells were treated with AgNPs or PBS, and were then trypsinized, washed twice in PBS, pelleted and stored at −20°C. Tumors and organs were minced and 200 mg of tissue was used for analysis. Samples were then digested with 10% HNO_3_ using a microwave‐assisted digestion system (Ethos UP, Milestone). The digested samples were diluted to a final acid concentration of 2% v/v for tumors and 1% v/v for organs before Ag determination by Inductively coupled plasma mass spectrometry (ICP‐MS). Trace metal grade HNO_3_ (Thermo Fisher Scientific), and distilled‐deionized water (18 MΩ cm, Milli‐Q^®^ (EMD Millipore)) were used to digest the samples and prepare all solutions. Standard reference solutions used for calibration were prepared in 2% acid (HNO_3_) for tumors or 1% acid for organs from a 1000 mg/L Ag stock (SPEX CertPrep, Metuchen, NJ, USA). A tandem ICP‐MS (8800 Triple Quadrupole; Agilent) equipped with a SPS 4 automatic sampler, a Scott‐type double pass spray chamber operated at 2°C, and a Micromist concentric nebulizer was used in all determinations. Helium gas (≥99.999% purity [Airgas]) was used in the ICP‐MS’s collision/reaction cell to minimize potential spectral interferences while monitoring the ^109^Ag isotope. Other relevant instrument operating conditions such as radio frequency applied power, sampling depth, carrier gas flow rate, reaction gas flow rate, and the number of sweeps per replicate were 1550 W, 10.0 mm, 1.05 L/min, 4.0 mL/min, and 100, respectively.

### 2.7 Transmission electron microscopy

Electron miocrographs of PVP‐stabilized, spherical 5, 25, 50, and 75 nm, or PVP, citrate, and silica shelled triangular silver nanoplates were provided by the manufacturer (nanoComposix Inc). Citrate stabilized, triangular silver nanoplates were dispersed in water and dried on copper‐coated formvar grids. MDA‐MB‐231, SUM159, iMEC, or MCF‐10A cells were grown in six‐well tissue culture dishes. Cells were treated with AgNPs (150 µg/mL) for 1 or 6 hours. All cells were washed thoroughly in PBS to remove AgNPs not bound or internalized by cells. After 1 hour, half of the wells were fixed in 2.5% glutaraldehyde at 4°C overnight. Fresh cell culture media was added to the remaining wells which were incubated for 5 hours more before fixation. Next, cells were scraped from the wells, pelleted, embedded in resin, cut into ultrathin sections (80 nm) and placed on copper‐coated formvar grids. All samples were imaged using an FEI Tecnai Spirit transmission electron microscope (Thermo Fisher Scientific). Samples were imaged without additional staining to facilitate the detection of AgNPs.

### 2.8 Western blots

Cells were grown on 10‐cm dishes at a density of 2 × 10^6^ cells in 10 mL of complete medium. Cells were allowed to recover for 18 hours and were then exposed to AgNPs for 6 or 24 hours at 37°C. Medium was removed, and lysates were collected using Mammalian Protein Extraction Regent supplemented with 1% Halt Protease & Phosphatase Inhibitor Cocktail (Thermo Fisher Scientific). Protein concentration was determined for each sample using a Pierce bicinchoninic acid protein assay kit (Thermo Fisher Scientific) according to the manufacturer's instructions. Proteins were size fractionated by gel electrophoresis and then transferred to a polyvinylidene fluoride (PVDF; Thermo Fisher Scientific) membrane. Nonspecific binding was blocked by incubation for 30 minutes at room temperature with tris‐buffered saline containing 5% powdered milk and 1% triton X‐100. Membranes were incubated overnight at 4°C with 1:1000 dilutions of primary antibodies (glucose regulated protein 78 (GRP78) (BiP; #3077), protein kinase RNA‐like endoplasmic reticulum kinase (PERK) (#5683), phospho‐alpha subunit of the eukaryotic translation initiation factor 2 complex (eIF2α) (#9721), eIF2α (#9722), CCAAT‐enhancer‐binding protein homologous protein (CHOP) (#2895), or β‐actin (#4970) purchased from Cell Signaling Technologies), washed, then incubated with anti‐rabbit (#7074) or anti‐mouse (#7076), horse radish peroxidase‐conjugated secondary antibodies also from Cell Signaling Technologies; (diluted 1:1000) for 1 hour at room temperature. Immunoreactive products were visualized by chemiluminescence (SuperSignal Femto West, Thermo Fisher Scientific) and quantified by densitometry using Bio‐Rad digital densitometry software.

### 2.9 Redox assays

Cells were plated and grown in clear bottom, white sided 96‐well plates. Reduced glutathione and oxidized glutathione were quantified using the Promega GSH‐Glo Glutathione Assay according to the manufacturer's instructions. Reduced nicotinamide adenine dinucleotide phosphate (NADPH) and its oxidized form were quantified using the Promega NADP/NADPH‐Glo Assay according to the manufacturer's instructions.

### 2.10 Flow cytometry

Cells (1.25 to 2.0 × 10^6^) cells were grown on 10 cm dishes. Cells were treated with AgNPs for 24 hours. Allophycocyanin (APC) annexin V (AnnV) and propidium iodide (PI) staining was performed as per the manufacturer's instructions (BD Biosciences). Labeled cells were analyzed on the Accuri6 Flow Cytometer (BD Biosciences). Analysis of data was performed using FCS Express version 3 (De Novo Software). Unstained controls were included to control for any potential interference of AgNPs with flow cytometry. For the AgNP doses tested (0‐150 μg/mL), there was no detectable change in forward, side scatter, PI fluorescence, or APC fluorescence in the unstained samples, indicating that AgNPs did not interfere with the assay.

### 2.11 Immunofluorescence

Cells were permeabilized for 20 minutes with 0.5% triton X‐100 in cytoskeleton buffer (100 mmol/L NaCl, 300 mmol/L sucrose, 10 mmol/L PIPES pH 6.8, 5 mmol/L MgCl2, 10 µg/mL aprotinin (all from Sigma‐Aldrich, St. Louis, MO, USA), 1 mmol/L 4‐(2‐aminoethyl)‐benzenesulfonyl fluoride, hydrochloride, and 250 µmol/L NaF (all from Roche Diagnostics, Risch‐Rotkreuz, Switzerland) and fixed with 4% paraformaldehyde. After blocking with 10% goat serum in immunofluorescence buffer (130 mmol/L NaCl, 13 mmol/L Na_2_HPO_4_, 3.5 mmol/L NaH_2_PO_4_, 7.7 mmol/L NaN_3_, 0.1% BSA, 0.2% triton X‐100, 0.05% tween 20), cells were incubated with the following antibodies (overnight, 4°C): 53BP1 (Ab36823; AbCam, Cambridge, UK; 5 µg/mL), γH2AX (clone JBW301; EMD Millipore, Burlington, MA; 2 µg/mL), Ki67 (PA5‐19462; Thermo‐Fisher Scientific; 1 µg/mL), β4‐integrin (MAB1964; EMD Millipore; 1:300), or ZO‐1 (clone 1A12, Invitrogen; 2.5 µg/mL). Primary antibodies were detected with secondary antibodies coupled to Alexa Fluor^®^ 488 or Alexa Fluor^®^ 568 or (Life Technologies; 1:500) incubated in blocking buffer 40 minutes at ambient temperature. DNA was counterstained with 4′,6‐diamidino‐2‐phenylindole (DAPI). Fluorescent signals were imaged with a Zeiss (Oberkochen, Germany) CLSM710 confocal microscope using a 63 × oil (NA = 1.4) objective. 53BP1 and γH2AX repair foci were quantified by visual scoring on confocal images (3D cultures). The percentage of S1 acini with apical polarity was determined by visual scoring of ZO‐1 signals using an Olympus IX83 microscope equipped with a 60× oil (1.35 NA) lens. A Cs‐137 irradiator (Mark I‐68A; JL Shepherd) was used for cell irradiation (3 Gy).

### 2.12 In vivo tumor treatment studies

All animal experiments were performed with prior approval by Wake Forest University Institutional Animal Care and Use Committee. Female, 8‐10 weeks old nu/nu mice were purchased from the Charles River Labs. Mice were housed in groups of five in individually ventilated cages with a 12 hours light/dark cycle and were allowed access to food and water ad libitum. Mice were acclimatized for 2 weeks prior to beginning experiments. MDA‐MB‐231 cells in growth factor reduced Matrigel^®^ (BD Biosciences) (50 µL containing 2 × 10^6^ cells) were injected into the fourth inguinal mammary fat pad of 15 mice. Tumor growth was monitored by calipers and volume was determined using the formula: volume = 0.52 × (width) × (length) × (width + length)/2 where length and width are the two largest perpendicular diameters. Tumors on 13/15 mice reached an average volume of 100 mm^3^ at approximately 3 weeks post‐implantation. These mice were weighed and randomized into two treatment groups (7 AgNP; 6 PBS). Mice were injected with AgNPs in PBS (6 mg/kg of silver) or PBS alone in the lateral tail vein (once for biodistribution or 3 times/week for 10 weeks for treatment). Tumor growth, body weight and general health were monitored over time.

### 2.13 Histology

Tumors were bisected, and fixed overnight in freshly prepared 4% paraformaldehyde in PBS. Tissue was paraffin embedded, sectioned, mounted on slides, and stained with hematoxylin and eosin. Slides were digitized at 63X magnification using a Hamamatsu Nanozoomer digital slide scanner and the digitized images were sent for pathology evaluation.

### 2.14 Statistical analysis

Analysis was performed as described in the figure legends using SigmaPlot 12.0 software (Systat Software Inc). IC_50_ for AgNP treatments was determined using Graphpad Prism 8 software (GraphPad Software).

## 3 RESULTS

### 3.1 Regardless of size, shape, or coating, silver nanoparticles selectively inhibit TNBC cell growth

**Figure 1:**
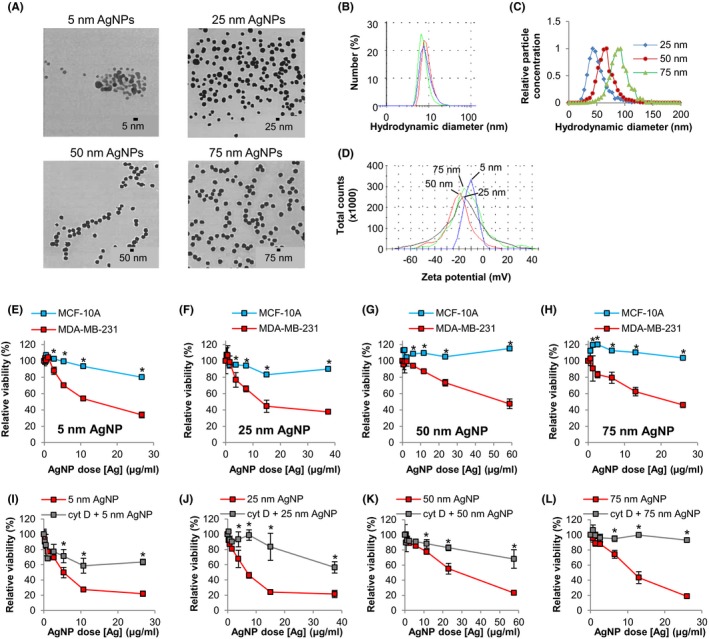
Determination of the effect of AgNP size and uptake on cytotoxicity in triple‐negative breast cancer and non‐malignant breast epithelial cells. Physicochemical characterization of AgNP prior to use in cell culture experiments included: (A) TEM imaging (provided by manufacturer [nanoComposix]); hydrodynamic size evaluation (B) of 5 nm AgNPs in triplicate using a Malvern Zetasizer NanoZS90 or (C) of 25, 50, and 75 nm AgNPs using a Nanosight NS500; (D) ζ‐potential measurement of 5, 25, 50, and 75 nm AgNPs. MDA‐MB‐231 and MCF‐10A cells were exposed to (E) 5 nm, (F) 25 nm, (G) 50 nm, or (H) 75 nm AgNPs for 48 h and viability was assessed by MTT assay. MDA‐MB‐231 cells were treated with (I) 5 nm, (J) 25 nm, (K) 50 nm, or (L) 75 nm AgNPs for 6 h in the presence of cytochalasin D to inhibit uptake. Viability was assessed by MTT assay 48 h after initial exposure. Data in E‐L represent means ± standard deviation from 6 technical replicates and are representative of duplicate independent experiments. Statistical analysis was performed by two‐way ANOVA and post‐hoc Tukey Test. Significant differences (*P < .05) are indicated

Initially, we used monodisperse AgNPs of increasing diameters (5, 25, 50, 75 nm) stabilized with a high (>74% by mass) percentage of PVP to determine if AgNP size influences the TNBC‐selective cytotoxicity we previously observed. 11 Free (ionic), silver measured by ICP‐MS was less than 1% of the total silver content according the manufacturer's specifications. These AgNPs were shipped from the manufacturer as dry powders, and aqueous dispersions in PBS were made freshly before use. Transmission electron micrographs of AgNPs show that the particles were homogeneous spheres (Figure 1A). We verified the manufacturer's data regarding particle size using dynamic light scattering (DLS) for 5 nm AgNPs (Figure 1B), or NTA for 25, 50, and 75 nm AgNPs (Figure 1C). All AgNPs were monodisperse. For each measurement, 50 μL of AgNP stock in PBS was diluted in 1 mL of the solution listed in the figure legends. This dilute concentration of PBS acted as a source of ions needed to form an electric double layer. All sizes of PVP‐coated, spherical AgNPs possessed negative ζ‐potentials at pH 6.5 (Figure 1D). We next exposed TNBC cells (MDA‐MB‐231) and non‐malignant mammary epithelial cells (MCF‐10A) to increasing concentrations of 5, 25, 50, or 75 nm AgNPs and evaluated viability 48 hours later (Figure 1E‐H). All sizes of AgNPs inhibited growth of MDA‐MB‐231 cells without significantly affecting MCF‐10A cells for all particle sizes at silver concentrations of 5 µg/mL or greater.

Next, we investigated whether cytotoxicity was dependent on uptake of AgNPs. Cyt D is a cell‐permeable actin depolymerizing agent that inhibits most energy dependent cellular uptake pathways including endocytosis, phagocytosis, pinocytosis, and caveolar uptake. We pulsed MDA‐MB‐231 cells with 5, 25, 50, or 75 nm AgNPs for 6 hours in the presence of Cyt D (37.5 nmol/L) to inhibit endocytosis, and then replaced the nanoparticle/drug containing media with fresh media. The cells were allowed to recover for 42 hours and viability was assessed. Cyt D significantly reduced AgNP‐induced growth inhibition (Figure 1I‐L), indicating that cytotoxic effects of AgNPs required internalization of the nanoparticles.

Subsequently, we used PVP‐stabilized, triangular silver nanoplates to determine if AgNP shape affected the TNBC‐selective cytotoxicity. The particles varied in size from approximately 75‐150 nm based on TEM, with a mean hydrodynamic diameter of 123 nm, and possessed a negative (−25 mV) ζ‐potential (pH 6.5) (Figure S1). Similar to what was observed for PVP‐stabilized, spherical AgNPs, PVP‐stabilized, triangular silver nanoplates at silver concentrations of 5 µg/mL or greater significantly inhibited growth of MDA‐MB‐231 cells but only modestly affected MCF‐10A cells (Figure S1). Notably, PVP‐stabilized, spherical gold nanoparticles (AuNPs; approximately 35 nm mean hydrodynamic diameter; −17 mV ζ‐potential in water [pH 6.5]) were not cytotoxic toward either MDA‐MB‐231 or MCF‐10A cells (Figure S2), which demonstrated that the TNBC selective cytotoxicity profile was not shared with PVP‐stabilized AuNPs.

We then synthesized chitosan coated, triangular silver nanoplates to determine if coating or surface charge affected the TNBC‐selective cytotoxicity of AgNPs. As shown in Figure S3, these nanoparticles were uniform in size and shape and possessed a mean hydrodynamic diameter of approximately 133 nm. In contrast to the negatively charged PVP‐stabilized particles, chitosan‐coated particles possessed a cationic (+27 mV) ζ‐potential at pH 6.5. As with PVP‐stabilized AgNPs, MDA‐MB‐231 cells were more sensitive than MCF‐10A cells to chitosan coated, triangular silver nanoplates at silver concentrations of 5 µg/mL or greater. No significant decrease in MCF‐10A growth was measured at any of the AgNP concentrations tested (Figure S3). To determine if a direct interaction between the AgNP surface and cell membranes or organelles was necessary for the TNBC specific cytotoxicity, we used triangular silver nanoplates encased in a silica shell to prevent direct binding to the silver NP surface. These silica‐shelled particles were similar in size (117 nm mean hydrodynamic diameter), ζ‐potential (−25 mV), and shape to the PVP‐stabilized triangular silver nanoplates (Figure S4). As with all other types of AgNPs, silica shelled, triangular silver nanoplates significantly inhibited the growth of MDA‐MB‐231 cells without affecting MCF‐10A cells at nanoparticle concentrations of 5 µg/mL or greater (Figure S4). Collectively, these results establish that the TNBC‐selective cytotoxic property of AgNPs requires internalization of the particles but is independent of particle size, shape, stabilizing agent, or surface charge.

**Figure 2:**
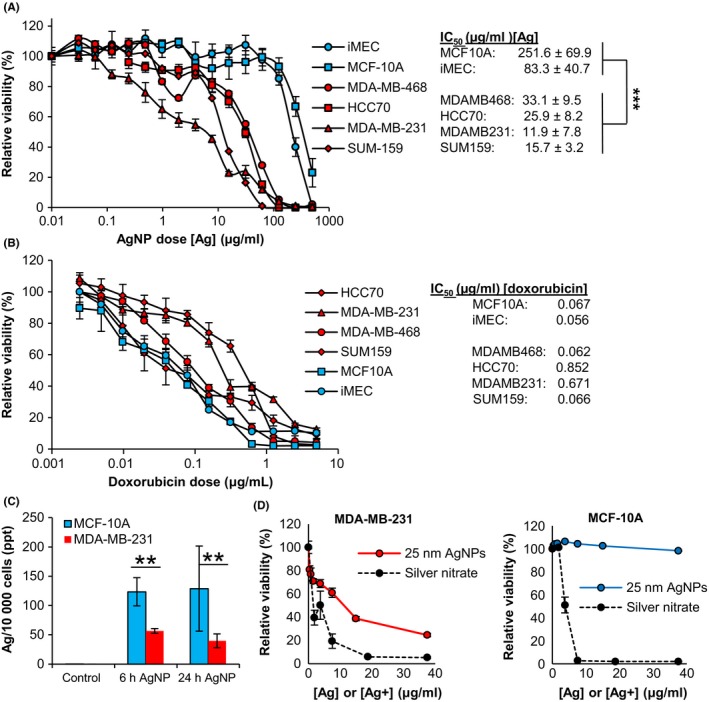
Assessment of the role of silver ion and reactive oxygen species in the cytotoxicity of AgNPs. A, Non‐malignant breast cells (white symbols) or TNBC cells (gray symbols) were exposed to 25 nm AgNPs for 72 h and viability was assessed by MTT assay. The IC_50_ of 25 nm AgNPs for each cell line is shown to the right. Data were obtained from 4‐6 technical replicates and 3 independent experiments depending upon cell line. Statistical analysis to compare IC_50_s for TNBC cells vs non‐malignant cells was performed by two‐way ANOVA and post‐hoc Tukey Test. Significant differences are indicated (***P < .001). B, Cells were exposed to doxorubicin for 72 h and viability was assessed by MTT assay. The IC_50_ dose of doxorubicin for each cell line is shown to the right. Data were obtained from 6‐8 technical replicates and are representative of duplicate independent experiments. C, Uptake of 25 nm AgNPs was quantified by ICP‐MS. Data were obtained from 3 technical replicates and duplicate independent experiments. Statistical analysis was performed by Student's T‐Test. Significant differences are indicated (**P < .01). D, Cells were exposed to 25 nm AgNPs or an equivalent silver concentration of AgNO_3_ for 48 h and viability was assessed by MTT. Data were obtained from 6 technical replicates and are representative of duplicate independent experiments

Based on these results, we selected 25 nm, PVP‐stabilized, AgNPs for further evaluation. We determined the cytotoxicity of this type of AgNPs in an expanded panel of breast cancer and non‐cancer cells, and the IC_50_ after 72 hours AgNP treatment was calculated for each cell line. All TNBC cell lines were significantly more sensitive to AgNPs than non‐malignant breast epithelial cell lines (Figure 2A). In contrast, doxorubicin, a chemotherapy drug commonly used to treat TNBC, was highly cytotoxic to non‐malignant breast epithelial cell lines (MCF‐10A, iMEC) and two of the TNBC cell lines (MDA‐MB‐468, SUM159) (Figure 2B). Two of the TNBC cell lines (MDA‐MB‐231, HCC70) were 10‐fold more tolerant to doxorubicin as compared to the non‐malignant breast epithelial cell lines. This indicates that increased sensitivity to AgNPs of TNBC cells vs non‐malignant breast cells is not due to a general sensitivity to conventional chemotherapy.

### 3.2 TNBC cells rapidly degrade silver nanoparticles after uptake

Having established that internalization of AgNPs was essential for their cytotoxicity, we quantified the cell uptake of AgNPs to determine if differences in uptake of AgNPs played a role in the sensitivity of TNBC cells to AgNPs. Based upon silver content, MCF‐10A cells took up almost twice as many AgNPs as MDA‐MB‐231 breast cancer cells, and most of the nanoparticles became cell associated during the first 6 hours of exposure (Figure 2C). Thus, the greater sensitivity of MDA‐MB‐231 cells to AgNPs was not due to taking up more AgNPs than the relatively insensitive MCF‐10A cells. We subsequently asked if a difference in sensitivity to Ag^+^ was responsible for the TNBC‐specific cytotoxicity of AgNPs. As shown in Figure 2D, Ag^+^ (AgNO_3_) was highly cytotoxic to both MDA‐MB‐231 and MCF‐10A cells. In contrast, MCF‐10A cells were tolerant to AgNPs, while MDA‐MB‐231 cells were sensitive to AgNPs.

We also characterized the time‐dependent colloidal stability of these AgNPs by DLS after hydration in water, PBS, and serum (Figure S5). No difference in hydrodynamic diameter (initially 44.4 ± 12.8 nm) indicative of aggregation was observed after dilution in water or PBS (pH 7.4) over 24 hours. When the serum concentration was increased to 55%, the mean particle size remained stable (34.9 ± 1.3 nm) over time. Lastly, we assessed the effect of storage conditions (temperature, light exposure) on AgNP integrity and cytotoxicity. Plasmon resonance frequency is dependent upon AgNP diameter, and thus is a sensitive metric to monitor particle dissolution. As shown in Figure S5, there was no change in the plasmon resonance frequency of spherical 25 nm, PVP‐stabilized, AgNPs after storage for 1 week in PBS in the light or dark at 4°C, room temperature, or 37°C. There also was no change in the cytotoxicity of AgNPs to MDA‐MB‐231 cells after storage of AgNPs under these conditions (Figure S5). These results indicate that these AgNPs did not substantially aggregate or degrade during storage.

**Figure 3:**
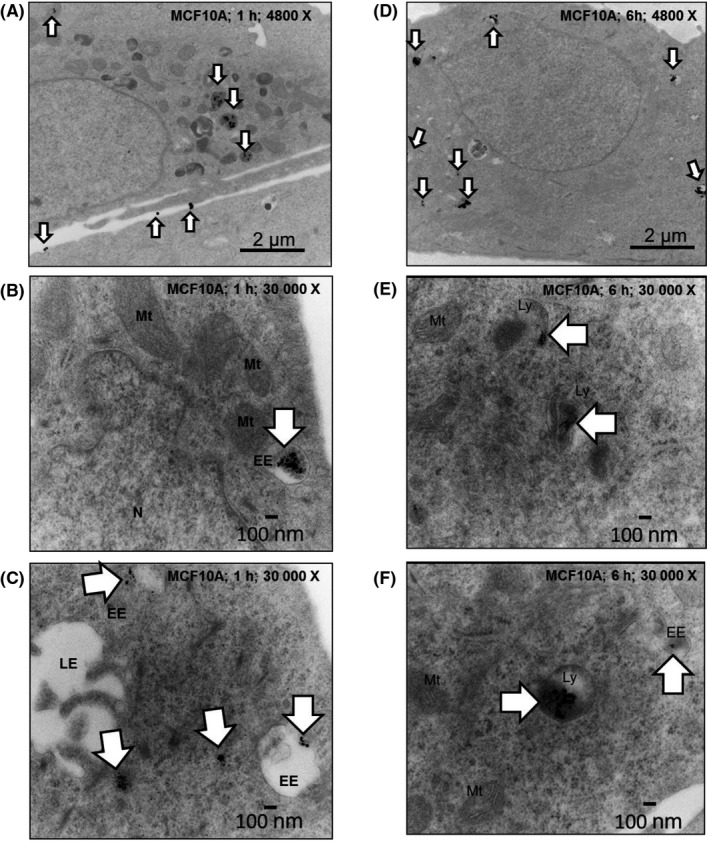
Imaging the uptake and trafficking of AgNPs in MCF‐10A cells. Electron micrographs show intact AgNPs in endosomes and cytoplasm (arrows) in MCF‐10A cells after a 1 h pulse at 4800 X magnification (A) or at 30 000 X magnification (B and C). AgNPs remain intact in lysosomes (arrows) after a 1 h pulse and 5 h chase cells at 4800 X magnification (D) or at 30 000 X magnification (E and F). Organelles and vesicles are identified in the images: EE, early endosome; LE, late endosome; LY, lysosome; Mt, mitochondria; N, nucleus

**Figure 4:**
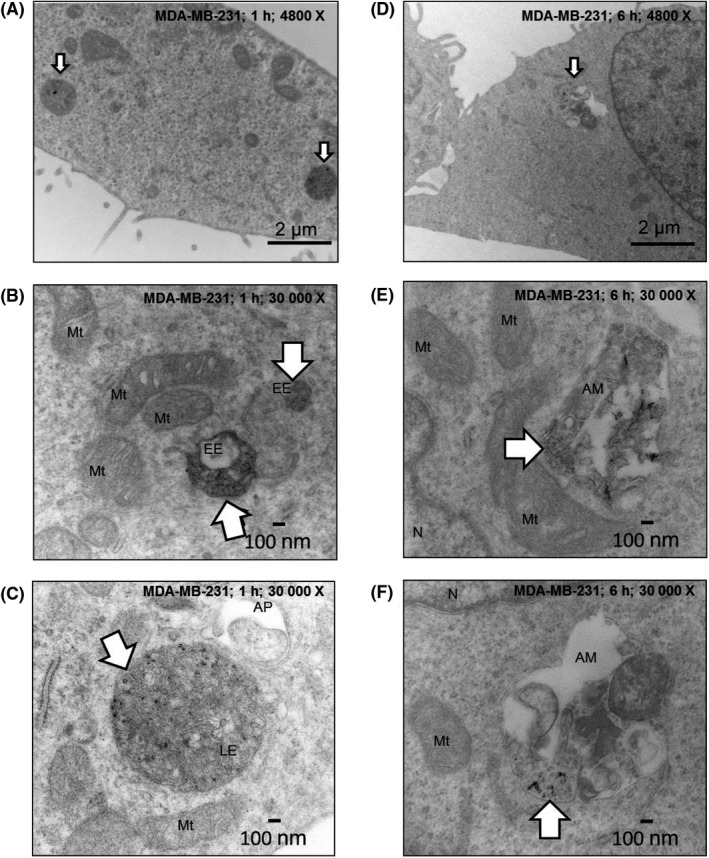
Imaging the uptake and degradation of AgNPs in MDA‐MB‐231 cells*.* Electron micrographs show degraded AgNPs in endosomes (arrows) of MDA‐MB‐231 cells after a 1 h pulse at 4800 X magnification (A) or at 30 000 X magnification (B and C). Degraded AgNPs are apparent in autophagic vesicles (arrows) after a 1 h pulse and 5 h chase cells in MDA‐MB‐231 cells at 4800 X magnification (D) or at 30 000 X magnification (E and F). Organelles and vesicles are identified in the images: AM, amphisome; AP, autophagosome; EE, early endosome; LE, late endosome; Mt, mitochondria; N, nucleus

We then imaged the uptake and intracellular distribution of AgNPs over time in MDA‐MB‐231 cells and MCF‐10A cells using TEM. Our images of AgNPs in MCF‐10A cells show intact nanoparticles were in endosomes after 1 hours, and intact AgNPs were still observed after these endosomes fused with electron dense lysosomes within 6 hours (Figure 3). After 1 hours, AgNPs were also found in endosomes of MDA‐MB‐231 cells, but the particles were notably degraded in comparison to those found in MCF‐10A cells, and after 6 hours, degraded AgNPs and damaged organelles were found in amphisomes (Figure 4), which are formed by fusion of late endosomes and autophagosomes. We performed a similar experiment in which the trafficking and degradation of AgNPs in iMEC cells and SUM159 cells were compared (Figures S6 and S7). After 1 hour, we observed AgNPs in endosomes in both cell lines. After 6 hours of exposure, we observed a high degree of degradation of AgNPs in the SUM159 cells, and in some cases, degraded AgNPs were co‐localized with damaged organelles in amphisomes. In contrast, AgNPs remained largely intact in iMEC cells. Thus, AgNPs are rapidly degraded and damage organelles in TNBC cells. In contrast, AgNPs are internalized, but remain intact in non‐malignant breast epithelial cells.

### 3.3 AgNPs delay progression through S‐phase, cause oxidative stress, ER stress, and apoptosis in TNBC cells without affecting non‐malignant breast epithelial cells

**Figure 5:**
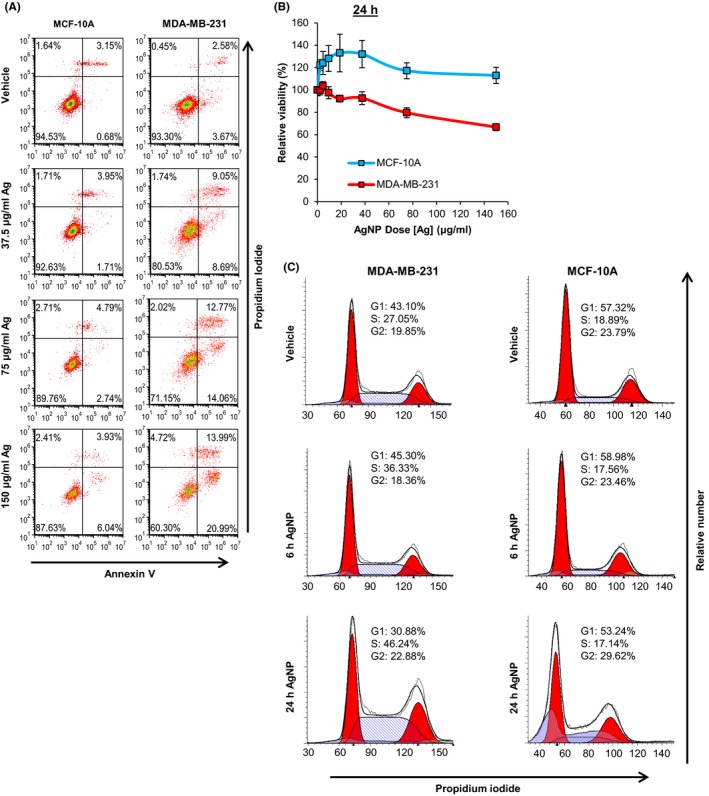
Assessment of the effect of AgNPs on cell cycle and cell death in MDA‐MB‐231 and MCF‐10A cells. A, MDA‐MB‐231 or MCF‐10A cells were treated with PVP‐stabilized, 25 nm AgNPs for 48 h, co‐stained with PI and AnnV, and then evaluated by flow cytometry. The percentages of cells characterized as viable (lower‐left quadrant), early apoptotic (lower‐right quadrant), late‐apoptotic (upper‐right quadrant), and necrotic (upper‐left quadrant) are shown within each quadrant. The presented data are representative of duplicate independent experiments. B, MDA‐MB‐231 or MCF‐10A cells were treated with 25 nm AgNPs for 24 h and viability was assessed by MTT assay. Data were obtained from 4‐6 technical replicates and 3 independent experiments depending upon cell line. C, MDA‐MB‐231 or MCF‐10A cells were treated with 37.5 µg/mL of 25 nm AgNPs for 6 or 24 h. Cells were fixed, permeabilized, and stained with PI, and then cell cycle analysis was performed by flow cytometry. The relative proportion of cells in each phase of the cell cycle is indicated. Sub‐G0/G1 cell populations indicative of apopotosis were excluded from the analysis

To determine if AgNPs induced cell death, AnnV and PI co‐staining was performed on the adherent population of non‐cancerous MCF‐10A breast cells and MDA‐MB‐231 cells treated with AgNPs for 48 hours. AgNPs induced a dose‐dependent increase in both early‐stage apoptosis and late‐stage apoptosis/necrosis in MDA‐MB‐231 (Figure 5A). Conversely, AgNPs had a minimal effect on early‐stage or late‐stage apoptosis/necrosis in MCF‐10A cells.

We then evaluated mechanisms of action and sought to identify potential sub‐lethal, on and off‐target toxicity of AgNPs. Although AgNP exposure was lethal to MDA‐MB‐231 cells after 48 hours (Figure 1) or 72 hours (Figure 2), a lesser effect on viability of MDA‐MB‐231 cells was observed after 24 hours (Figure 5B). Therefore, at this early time point, it was possible to examine sub‐lethal effects of AgNPs that contributed to cell death at subsequent time points. We initially examined the effect of AgNP treatment on the cell cycle to determine if AgNPs also induced growth arrest in addition to cell death (Figure 5C). Treatment of MDA‐MB‐231 cells with AgNPs (37.5 μg/mL) induced a time‐dependent decrease in the number of cells in G0/G1 and an increase in S‐phase cells. In contrast, there was little effect on the cell cycle distribution of MCF‐10A cells treated with AgNPs.

**Figure 6:**
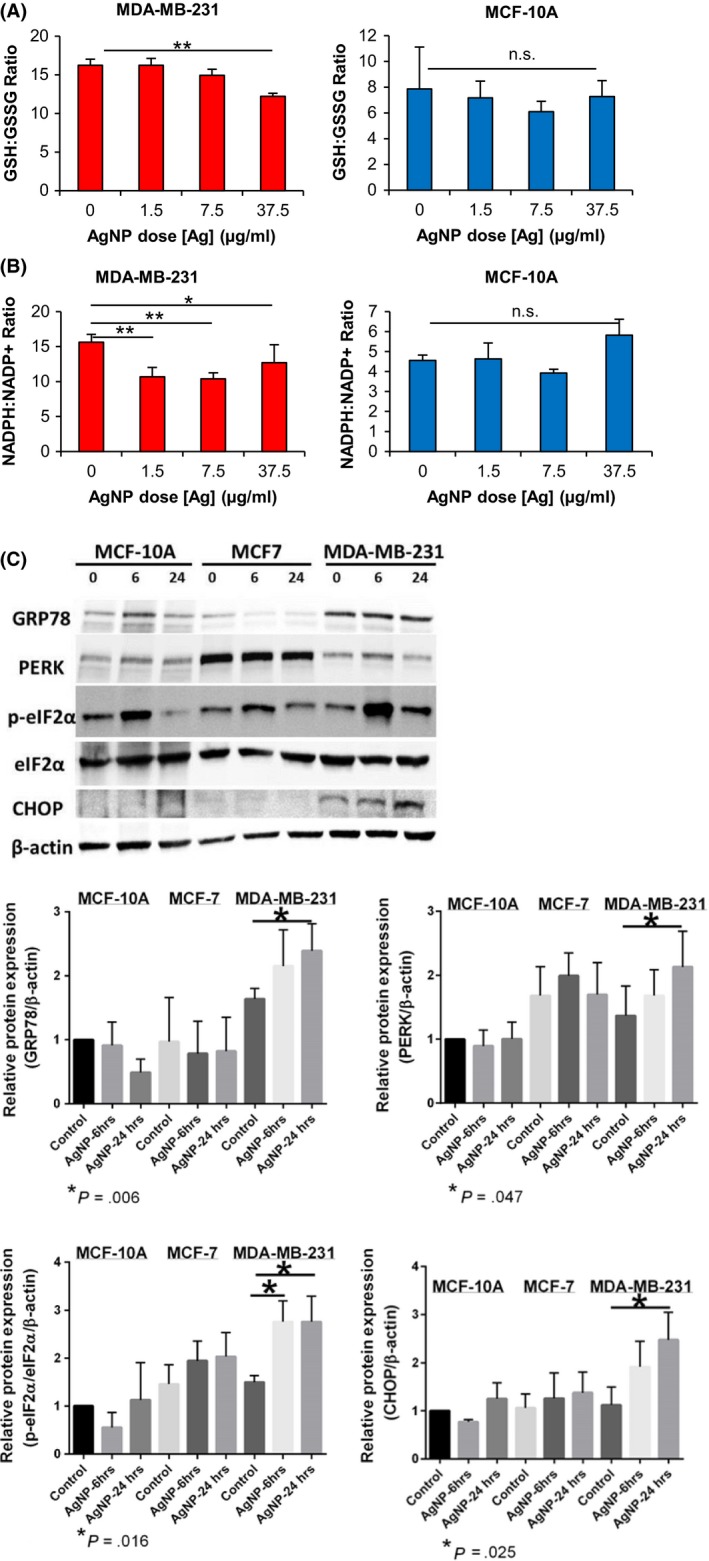
Quantification of oxidative and ER stress in triple‐negative breast cancer and non‐malignant breast cells*.* The ratios between (A) reduced and oxidized glutathione (GSH/GSSH) or (B) reduced and oxidized nicotinamide adenine dinucleotide phosphate (NADPH/NADP^+^) were quantified in cell lysates following sublethal exposure of MDA‐MB‐231 or MCF‐10A cells to AgNPs for 24 h. Data were obtained from 4 technical replicates and are representative of duplicate independent experiments. Statistical analysis was performed by one or two‐way ANOVA and post‐hoc Tukey Test. Significant differences between treatment groups are indicated (*P < .05; **P < .01; NS, non‐significant (ANOVA, *P* > .05)). (C) MDA‐MB‐231, MCF‐7, and MCF‐10A were treated with AgNPs for 6 or 24 h, and then cell lysates were analyzed for markers of ER stress and activation of the unfolded protein response by western blot, as indicated. Representative western blots show that AgNPs induce ER stress in MDA‐MB‐231 cells but not in MCF‐7 or MCF‐10A cells. Protein levels relative to the β‐actin loading control were quantified by densitometry. Expression of PERK, p‐eIFα/total eIFα, GRP78, and CHOP is shown relative to levels detected in untreated MCF‐10A cells. Data were obtained from 5 independent experiments. Statistical analysis for each cell line was performed by one‐way ANOVA and post‐hoc Student's *t* test. Significant differences (*P < .05) in protein levels relative to baseline for each cell line are shown with *P*‐values indicated in each panel

Subsequently, we quantified the effects of 24 hours AgNP exposure on cellular redox balance. The tripeptide non‐protein thiol, glutathione (GSH), plays a key role in mitigating oxidative damage. In the presence of reactive oxygen species (ROS), GSH is oxidized to form a homodimer disulfide (GSSG). NADPH also protects against oxidative stress and provides reducing equivalents allowing the regeneration of reduced GSH from its oxidized disulfide form (GSSG). Therefore, substances causing imbalances in the redox balance of GSH/GSSG and NADPH/NADP^+^ may impact normal cell function, even at non‐lethal doses. To determine the effect of AgNPs on the redox state of MDA‐MB‐231 and MCF‐10A cells, we quantified the ratio of the oxidized and reduced forms of these antioxidants before and after cells were exposed to AgNPs. AgNP treatment decreased the GSH/GSSG ratio in MDA‐MB‐231 cells, but not in MCF‐10A cells (Figure 6A). Similarly, AgNP treatment also decreased the NADPH/NADP^+^ ratio in MDA‐MB‐231 cells, but not in MCF‐10A cells (Figure 6B).

We previously observed that uncoated AgNPs were less cytotoxic to MCF‐7 cells (luminal A breast cancer, non‐TNBC) than to TNBC cells,^11^ and we verified that this was also true for the PVP stabilized, 25 nm AgNPs used in this study (Figure S8). We then treated MDA‐MB‐231, MCF‐7, and MCF‐10A cells with AgNPs overnight to see if AgNPs induced ER stress and UPR in TNBC cells without affecting non‐malignant cells, and to determine if TNBC cells were more sensitive to AgNP induced ER stress than luminal A breast cancer cells. We quantified total PERK (protein kinase R‐like ER kinase) and GRP78 (78 kDa glucose‐regulated protein) in MCF‐10A, MCF‐7, and MDA‐MB‐231 cells after 6 or 24 hours exposure to AgNPs (37.5 μg/mL). No significant change in GRP78 or PERK was found for MCF‐10A or MCF‐7 cells, indicating that ER stress was not induced at this dose of AgNPs (Figure 6C). However, both PERK and GRP78 were significantly increased in MDA‐MB‐231 cells. Activation of the PERK signaling cascade initiates the UPR through phosphorylation of eukaryotic translation initiation factor 2α (eIF2α or p‐eIF2α when phosphorylated). Failure to mitigate ER stress leads to synthesis of the pro‐apoptotic protein, CHOP (C/EBP homologous protein). After exposure to AgNPs, no significant change in p‐eIF2α/total eIF2α ratio or CHOP expression was found for MCF‐10A or MCF‐7 cells, indicating that the UPR was not induced (Figure 6C). In contrast, AgNPs activated the UPR in MDA‐MB‐231 as indicated by increases in both the p‐eIF2α/eIF2α ratio and CHOP expression. Increased CHOP expression in MDA‐MB‐231 cells treated with AgNPs is expected to induce apoptosis, which is consistent with the results shown in Figure 5A.

### 3.4 AgNPs cause DNA damage and apoptosis in 3D cell culture models of TNBC but do not damage 3D culture models of the normal mammary gland

**Figure 7:**
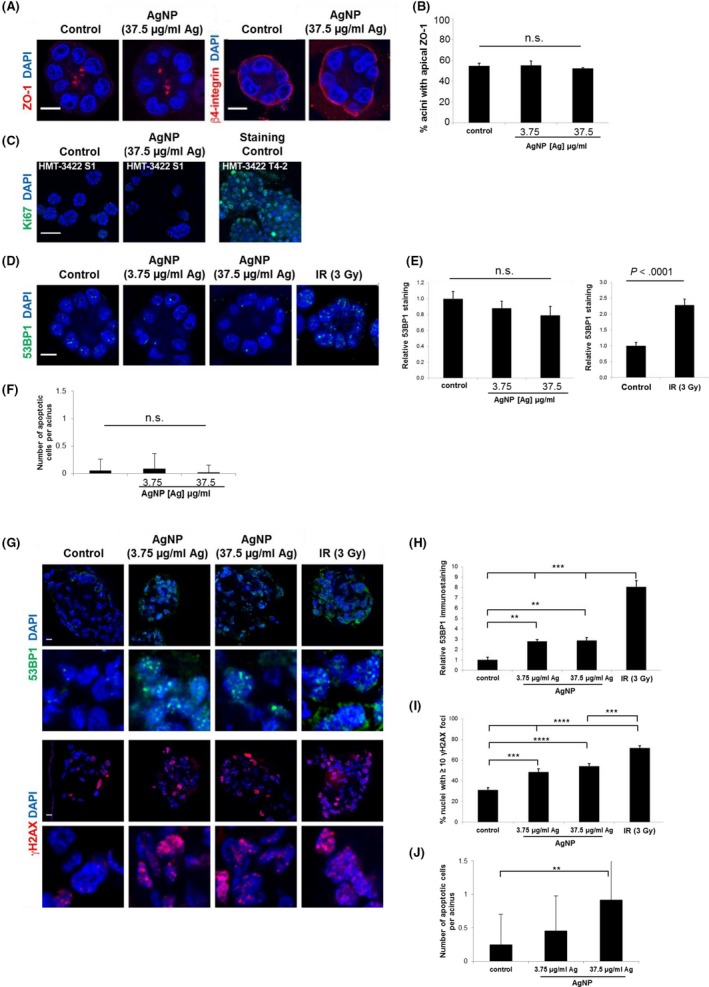
Quantification of DNA damage and apoptosis in triple‐negative breast cancer tumor nodules and non‐malignant breast cells grown in 3D cell culture. A, Representative confocal images of non‐malignant S1 acini treated for 48 h with 3.75 or 37.5 μg/mL of AgNP or with PBS (control) and immunostained for the tight junction marker ZO‐1 or the basal marker β4 integrin. Nuclei were counterstained with DAPI. B, Quantification of apical ZO‐1 localization in acini treated as in (A). Mean ± standard error from 3 independent biological replicates are shown. At least 100 structures were scored per condition for each replicate. No significant differences were detected between treatment groups (NS; ANOVA, *P* > .05). C, Staining for the proliferation marker Ki67 in S1 acini differentiated in 3D culture. Ki67 staining was validated by parallel analysis of S1‐derived T‐42 breast cancer cells. D, Detection of DNA damage by immunostaining for 53BP1 in S1 acini treated with AgNP or PBS. Exposure of acini to ionizing radiation (3 Gy, IR) was used for validation. E, For each acinus cross‐section, the average number of 53BP1 foci/nucleus in confocal images of S1 acini was quantified to determine if DNA damage was induced by AgNP exposure. The bar graph represents mean ± standard error (N > 20 acini from two independent biological replicates) after normalization to PBS‐treated cells. No significant differences between AgNP treatment groups was detected (NS; ANOVA, *P* > .05). However, significant differences (*t* test; *P* < .0001) in 53BP1 foci were detected between acini exposed to IR or mock‐irradiated. F, The number of apoptotic cells per acinus was estimated based on pyknosis and karyorrhexis detected with DAPI staining of S1 acini treated as in (A). No significant differences in between treatment groups were detected (ANOVA; *P* > .05; N > 20 acini from two independent biological replicates). Scale bars = 10 µm. G, Detection of 53BP1 (green) and phosphorylated H2AX (γH2AX, red) by confocal microscopy in MDA‐MB‐231 cells cultured in 3D with Matrigel. Cells were treated for 48 h with PBS (control) or AgNPs. Exposure to 3 Gy of ionizing radiation (IR) served as positive control for DNA damage detection. Scale bars = 10 µm. Zoomed images are shown in the lower panels for each stain. H, For each nodule cross‐section of MDA‐MB‐231 cells treated as in (G), the average number of 53BP1 foci/nucleus was scored. Means ± standard error are shown after normalization to control. Significant differences between treatment groups were detected as indicated (ANOVA; **P < .01 and ***P < .001; N ≥ 7 nodules from two independent biological replicates). I, The proportion of nuclei with at least 10 γH2AX foci per cross‐section in MDA‐MB‐231 cells treated as in (G). Significant differences between treatment groups were detected as indicated (ANOVA; ***P < .001 and ****P < .0001; N = 9 nodules from two independent biological replicates). J, The number of apoptotic cells per nodule was estimated based on pyknosis and karyorrhexis detected with DAPI staining in confocal images of MDA‐MB‐231 tumor nodules treated as in (G) and significant differences in between treatment groups were detected as indicated (ANOVA and post‐hoc Tukey test; ** *P* < .01; N = 9 nodules from two independent biological replicates). Scale bars in A, C, D, G = 10 µm

We exposed three dimensional cultures of non‐neoplastic S1 mammary epithelial cells (S1 cells) to AgNPs for 48 hours to assess if AgNPs affected the normal mammary gland architecture. S1 cells grown on Matrigel^®^ develop growth‐arrested, polarized spherical structures similar to breast acini. S1 acini treated with AgNPs retained their characteristic single‐layer spherical organization, with no multilayering or detectable disorganization, as observed with DAPI staining. AgNP exposure did not disrupt apical localization of the tight junction marker ZO‐1 or basal localization of β4 integrins (Figure 7A,B). A lack of positivity for Ki67 staining indicated that AgNP treatment did not induce proliferation of S1 cells (Figure 7C), nor did AgNP treatment induce detectable levels of DNA damage, visualized by 53BP1 staining (Figure 7D,E). Treatment of S1 cells with ionizing radiation was used to validate the detection of DNA damage. Scoring pycnotic and karyorrhectic nuclei in S1 acini revealed no increase in apoptosis for AgNP‐treated cells (Figure 7F). AgNPs were also not cytotoxic to S1 cell monolayers (Figure S8).

We then grew MDA‐MB‐231 cells in three‐dimensional cultures as above. Under these culture conditions, MDA‐MB‐231 cells do not growth‐arrest and develop disorganized masses reminiscent of tumor nodules. We then exposed the MDA‐MB‐231 tumor nodules to AgNPs, using the same doses as for S1 acini. Both AgNP concentrations induced a significant increase in 53BP1 and γH2AX DNA repair foci in MDA‐MB‐231 cells compared to control, indicating DNA damage induction by AgNPs (Figure 7G‐I). Scoring pycnotic and karyorrhectic nuclei in MDA‐MB‐231 tumor nodules revealed increased apoptosis in AgNP‐treated cells (Figure 7J).

### 3.5 Intravenous delivery of AgNPs is effective for treatment of TNBC xenografts in vivo

We examined the potential to use AgNPs for in vivo treatment of tumors. First, we conducted dose escalation studies in mice to establish a safe dosing range. Groups of five mice were injected intravenously with a single dose of AgNPs (6, 9, or 12 mg total silver/kg body weight) and mice were monitored for signs of distress (weight loss; dehydration; rapid or shallow breathing; hunched posture/immobility; piloerection; guarding behavior; bleeding from any orifice; death). All mice in the 6 mg/kg group tolerated the dose with no apparent toxicity. Two mice in the 9 mg/kg group exhibited signs of distress 24‐48 hours after injection (shallow breathing; hunched posture/immobility), but recovered within a few days. All mice in the 12 mg/kg group showed signs of severe toxicity within 24 hours (dehydration; rapid or shallow breathing; hunched posture/immobility) and 2 mice died after 48 hours. Based upon this data, we selected 6 mg/kg for subsequent biodistribution and tumor treatment studies.

**Figure 8:**
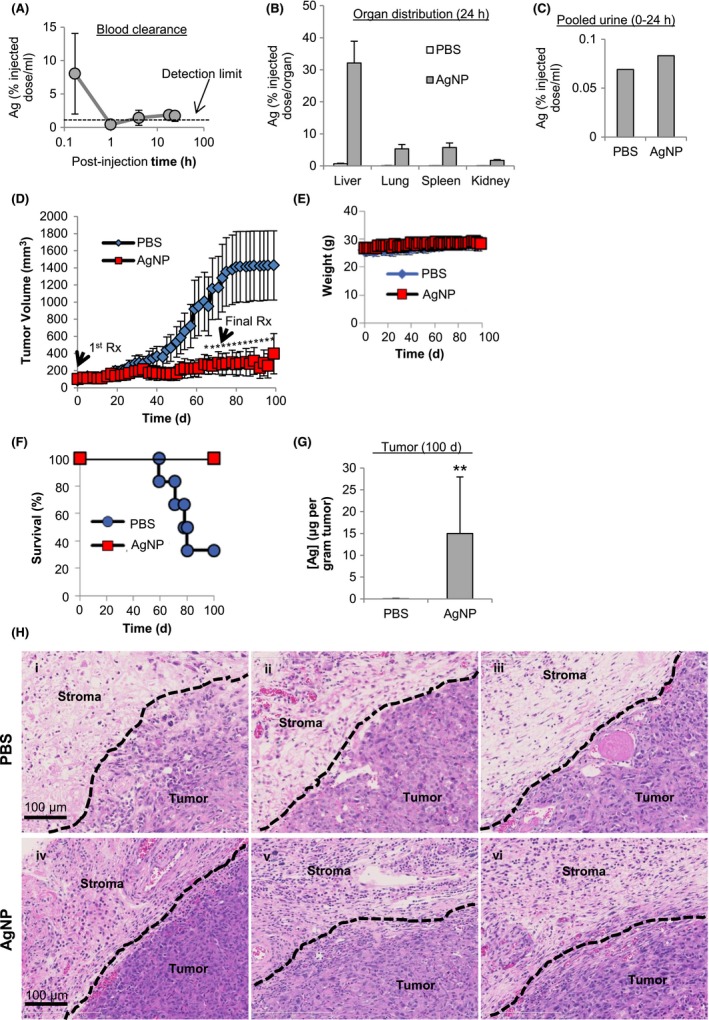
Safety and efficacy of intravenous delivery of AgNPs for treatment of triple‐negative breast cancer tumors in vivo. (A) Blood clearance, (B) biodistribution, and (C) urine content of AgNPs (6 mg/kg; single dose; IV) were quantified in nude mice by ICP‐MS after 24 h for 20 µL of blood, the entire organ, or entire volume of collected urine. (D) MDA‐MB‐231 tumor bearing nude mice were intravenously injected with PBS (6 mice) or AgNPs (7 mice; 6 mg/kg; 3× per wk; 10 wks) and tumor growth was quantified over time by calipers. (E) Mice were monitored for weight change. Statistical analysis was performed using two‐way ANOVA and post‐hoc Tukey test. Significant differences in tumor size are indicated (*P < .05). (F) Survival of treated mice is plotted by Kaplan‐Meyer analysis. Due to tumor growth in excess of the severity limit of the protocol (>1000 mm^3^) 2/3 of PBS treated mice were euthanized prior to the end of the study at 100 d. No tumors in AgNP treated mice reached the tumor size limit and all mice survived until the completion of the study at 100 d. (G) Silver content in residual tumors of AgNP treated mice and PBS treated mice was quantified by ICP‐MS. Statistical analysis was performed by student *t*‐test. A significant difference in silver content is indicated (**P < .01). (H) Hematoxylin and eosin stained sections of tumor and adjacent stroma from individual PBS (panels i‐iii) and AgNP (panels iv‐vi) treated mice are shown

The blood clearance profile following a single intravenous injection of AgNPs indicated that AgNPs were cleared from the circulation within 1 hours of administration (Figure 8A). After 24 hours, the largest amount of silver was in the liver, with lower detectable levels found in the lungs, spleen, and kidneys (Figure 8B). Minimal silver was excreted in the urine (Figure 8C). Next, nude mice bearing orthotopic, MDA‐MB‐231 tumors were injected intravenously, three times per week for 10 weeks, with AgNPs (6 mg total silver/kg per injection) or PBS. We found that AgNPs significantly reduced MDA‐MB‐231 tumor growth in mice (Figure 8D). No difference in weight between PBS and AgNP treated mice was observed (Figure 8E), nor were there overt signs of distress (described above), indicating that this dose of AgNPs was potentially both effective and safe. All mice treated with AgNPs (7/7) survived for the duration of the study (100 days) while only one third of the PBS group (2/6) survived (Figure 8F).

After 100 days, all AgNP and remaining PBS treated animals were euthanized. An average of approximately 15 μg of silver per gram of tissue was detectable within the tumor/surrounding necrotic tissue of mice treated with AgNPs (Figure 8G). To determine if AgNPs affected tissue and stroma neighboring the tumor target, a blinded pathologist examined the histology of tumors from PBS and AgNP treated mice. The pathology report indicated the presence of viable tumor and areas of necrosis in all samples, but the residual tumors were significantly smaller in AgNP treated mice than in PBS treated mice. The stroma adjacent to tumors of all AgNP treated mice had a notable increase in cellularity due to the presence of increased numbers of immune cells, especially plasma cells, that were absent in PBS‐treated mice (Figure 8H).

## 4 DISCUSSION

Here, we show for the first time that systemically administered AgNPs are effective for reducing the growth of solid, TNBC mammary tumors in mice, which supports the possibility that AgNPs may be useful for treatment of some human breast cancers. Notably, doses of AgNPs that are cytotoxic to TNBC cells in vitro do not cause toxicity to, or disrupt the homeostasis of non‐malignant breast epithelial cells. We establish that the TNBC‐selective cytotoxic property of AgNPs is dependent upon exposure of cells to intact AgNPs, but is independent of particle size, shape, or capping agent. Because the TNBC‐selective cytotoxicity of AgNPs is not shared by ionic silver, it can be considered a “new to nano” cytotoxic property. The AgNPs used for the majority of our studies consist of only two components: silver and a dense stabilizing layer of PVP, a polymer considered generally safe by the United States Food and Drug Administration. When added to existing facile and scalable production capabilities, this simplicity makes them attractive for further development.

Due to the heterogeneity of human tumors, differences in drug dosing and pharmacokinetics between mice and humans, and the potential for unexpected toxicities, a significant knowledge gap exists when extrapolating from cell lines and animals to humans. However, preclinical cancer models represent an important tool for the development of new cancer therapies, and use of multiple cell culture and mouse models of human cancer is the most likely method to predict the efficacy of novel anti‐cancer treatments.^45^ To address the heterogeneity of TNBC, we used four TNBC cell lines representing both basal A (HCC70, MDA‐MB‐468) and basal B (MDA‐MB‐231; SUM159) subtypes.^46^ Basal A cell lines correspond to the breast cancer molecular phenotype identified by Perou et al as basal‐like, and basal B cell lines are similar to claudin‐low breast tumors.^46^ However, Pietenpol et al proposed the existence of as many as 6 subtypes of TNBC,^47^ and not all potential molecular subtypes of TNBC were assessed in our study. Nonetheless, we observed high cytotoxicity of AgNPs in all four immortalized TNBC cell lines.

In addition, we found that the cytotoxicity of AgNPs was significantly less in three different non‐tumorigenic breast epithelial cell lines commonly used as models for normal breast epithelial cells. Both the MCF‐10A and HMT‐35522‐S1 cell lines were derived from benign mammary fibrocystic lesion and were immortalized spontaneously through culturing on plastic.^48^, ^49^ The iMEC cell line was derived from primary mammary epithelial cells that were immortalized by retroviral transduction with cDNA for the H‐catalytic subunit of human telomerase (hTERT).^50^ Furthermore, in a previous study,^11^ we found that both non‐malignant 184B5 breast epithelial cells, which were immortalized by chemical transformation of mammary tissue obtained from a normal reduction mammoplasty,^51^ and primary, non‐transformed, mammary epithelial cells were approximately tenfold less sensitive to uncoated AgNPs than TNBC cell lines.^11^ Although no single breast cell line or model system is a perfect representation of the normal breast epithelium or TNBC, and MCF‐10A cells may exhibit growth characteristics that are inconsistent with normal breast epithelial cells under some culturing conditions,^52^ it is unlikely that our data, which show that TNBC cell lines are more sensitive to AgNPs than non‐malignant breast cells, are simply coincidental based upon cell line selection. We also used 3D organoids and murine xenografts to evaluate treatment of TNBC with AgNPs, and to identify potential off‐target effects of AgNPs. Regardless of the model used, we consistently observed that AgNPs damaged TNBC cells and tumors at doses that did not harm the non‐malignant cell types that were examined.

For clinical development of AgNP‐based therapeutics, it will be necessary to define the specific physicochemical features of the nanoparticles that will be used, and to tie these properties to biological outcomes. Comparative evaluation of nanoparticle toxicity is challenging because factors that affect physicochemical features such as particle size, ζ‐potential, and reactivity can also influence colloidal properties, which in turn affect solution dynamics, cell uptake, intracellular trafficking, and exposed dose. The difficulty of identifying which factor contributes to a particular toxicity profile is daunting, and likely plays a role in the lack of reproducibility of many of the studies that attempt to do so.^53^ To avoid potentially misleading conclusions following exposure of individual cell lines to different types of AgNPs, we initially assessed differences in the response of both TNBC and non‐malignant cell lines to identical AgNPs to identify aspects of the AgNP cytotoxicity profile that are dependent upon the underlying biology of the cell target rather than differences in physicochemical properties of nanoparticles (Figure 1 and Figures S1‐S4). Notably, we also find that the TNBC selective cytotoxicity of AgNPs is conserved across a variety of AgNPs with different sizes, shape, or coating. Although the high cytotoxicity of AgNPs in TNBC and low cytotoxicity in non‐malignant breast cells are unaffected by changing sizes, shape, or coatings, it is important to note that uptake and dissolution rates, pharmacokinetics, and biodistribution of AgNPs are dependent upon these properties, and this will affect the magnitude and timing of the response of cells and tumors to AgNPs. These issues were not investigated here. Further research is needed to identify specific AgNP characteristics that enhance cytotoxicity in TNBC cells, increase tumor accumulation, and improve body clearance of AgNPs without increasing off‐target toxicity.

Despite these limitations, we demonstrate effective treatment of a TNBC tumor in vivo at an AgNP dose that did not cause overt toxicity in mice (Figure 8). It is likely that the silver still found in the tumors 30 days after treatment cessation (Figure 8G) is in the form of silver sulfides, which are insoluble and can be retained in humans for long periods of time without toxicity.^54^ The main cause for concern from such compounds is argyria, a discoloration of the skin that can be treated with dermal lasers.^55^ However, no color change indicative of argyria was observed for the mice in our study. Our finding regarding the lack of overt in vivo toxicity of 25 nm, PVP‐stabilized AgNPs at a repeated, 6 mg/kg IV dose is in agreement with comprehensive toxicity studies in rodents previously performed using PVP‐stabilized AgNPs with similar characteristics to our AgNPs. In one study, rats received 28 daily, intravenous injections of AgNPs at a dose of 6 mg/kg, and no dose limiting toxicity was observed, though transient effects on liver and immune cell function were noted.^56^ Additionally, similar AgNPs to the ones used in our studies did not affect platelet aggregation, coagulation, or complement activation in mice.^57^ Detailed metabolomics studies following IV injection of mice with 30 nm, PEG‐thiol‐coated AgNPs (8 mg/kg) also showed no evidence of gross toxicity after this dose, but modest effects on liver function were observed.^58^ These effects appeared transient and were not believed to be indicative of persistent liver injury.

Single‐blind human safety studies reported that orally‐dosed colloidal silver supplements (which contain AgNPs) did not cause detectable changes in metabolic, hematologic, or urinalysis measures, inflammatory cytokine secretion, ROS generation, or morphological changes in the lungs, heart or abdominal organs,^59^ and no changes in platelet function were noted.^60^ Although the reported toxicity in humans of colloidal silver supplements taken orally was negligible in clinical trials, the largest dose tested (480 μg per dose; equivalent to a dose of 6.4 μg/kg for a 75 kg human) was almost 1000‐fold less than the dose used for our studies in mice (6 mg/kg). In addition, the material used in our studies contained less than 1% Ag^+^ by weight and was delivered IV, but the products tested in these clinical studies contained as much as 84% dissolved Ag^+^ by weight, and systemic bioavailability of this material following oral ingestion was extremely low.

The results of our in vitro mechanistic and safety studies (Figures 5, 6, 7) showed that doses of AgNPs that damage TNBC cells and 3D tumor nodules in vitro do not affect the cell cycle, impair redox homeostasis, induce ER stress, cause DNA damage, or induce apoptosis in non‐malignant, breast epithelial cells, nor do they affect cell polarity in our model of the normal mammary gland. The normal breast epithelium consists of glandular structures (acini) connected to a branched ductal system. The architecture of the acini and the ducts is characterized by a central lumen, apical and lateral cell‐cell junctional complexes (including apical tight junctions), and hemidesmosomes ligating the basement membrane at the basal side of the gland/duct.^61^ Establishment and maintenance of apical‐basal polarity is essential for homeostasis, and loss of polarity is linked to breast cancer initiation.^62^, ^63^

A recent study showed that non‐TNBC, luminal A breast cancer cell lines (MCF‐7 and T‐47D) were more sensitive to AgNP‐induced ER stress than non‐malignant, MCF‐10A cells.^64^ We found that MDA‐MB‐231 cells were even more sensitive to AgNP‐induced ER stress than MCF‐7 cells (Figure 6C,D). Several of the AgNP sensitive, TNBC cell lines we identified are also reported to constitutively activate the PERK‐eIF2α axis of the UPR to deal with the stress of protein synthesis, and these cells are highly sensitive to agents that further induce ER stress.^38^ We focused only on the PERK arm of the UPR. However, ER stress can activate three arms of the UPR, each of which is referred to by its initiating stress sensor, which include inositol‐requiring protein 1 (IRE1) and activating transcription factor‐6 (ATF‐6) in addition to PERK. There are conflicting reports on activation of the IRE1 arm by AgNPs with one study indicating its activation following AgNP exposure^36^ and another showing no change.^65^ Less is known about the role of the ATF‐6 arm following AgNP exposure, but AgNPs reportedly degrade ATF‐6 in some cell lines.^65^ While more research is needed to fully define the importance of each arm of the UPR in mediating the response to AgNPs, our results provide evidence that induction of ER stress by AgNPs is a potentially exploitable vulnerability for treatment of TNBC.

We also found that the percentage of MDA‐MB‐231 cells, but not MCF‐10A cells, in S phase increases after AgNP treatment. However, ER stress and the UPR are expected to induce G1^66^ or G2 arrest.^67^ Activation of p53 is needed for G2 arrest,^67^, ^68^ Additionally, AgNPs induce DNA damage in these cells, and the slowing of progression through S‐phase after AgNP exposure may occur as cells attempt to repair damaged DNA.^69^ The mutant p53 status of MDA‐MB‐231 cells may affect how the cells respond to DNA damage, and alter the cell cycle after AgNP exposure. We did not specifically assess the role of p53 in sensitivity to AgNPs as almost all TNBC cell lines (including the 4 used in our study) are p53 mutants, and up to 80% of TNBC patients have mutant p53.^70^ However, a previous report has shown that AgNPs are able to induce p53‐independent cancer cell apoptosis in both p53 wild type and p53‐mutant osteosarcoma cells.^71^

The induction of TNBC cell death following exposure to AgNPs appears to be delayed. As shown in Figure 5B, AgNPs cause little change in MDA‐MB‐231 viability 24 hours after initial exposure, but viability decreases substantially after 48 hours (Figure 1E‐H), and even more so after 72 hours (Figure 2E). Because the total amount of AgNPs associated with the cells did not change between 6 and 24 hours (Figure 2A), the increased loss of viability does not appear to be due additional uptake of AgNPs over time. Consistent with this, MDA‐MB‐231 cells that were exposed to AgNPs continuously for 48 hours (Figure 1E‐H) exhibited a similar loss of viability to cells that were exposed to AgNPs for only 6 hours (Figure 1I‐L) when viability was assessed 48 hours after the initial exposure. This supports the idea that at the doses tested, AgNPs cause an accumulation of damage that leads to cell death through ER stress and DNA damage rather than causing acute, catastrophic damage to cell membranes.

The precise reason why TNBC cells are inherently more sensitive to AgNPs than non‐malignant breast epithelial cells remains to be determined. The cytotoxicity of AgNPs is believed to involve the release of silver cation (Ag^+^).^24^, ^72^, ^73^, ^74^, ^75^ We find that both MCF‐10A and MDA‐MB‐231 cells are sensitive to exposure to Ag^+^ (Figure 2C), but our studies suggest that MDA‐MB‐231 cells degrade AgNPs more rapidly than MCF‐10A cells (Figures 3 and 4). Similarly, we find that AgNPs are more rapidly degraded in SUM159 cells as compared to iMEC cells (Figures S6 and S7). Thus our data support a mechanism whereby the greater rate of intracellular release of Ag^+^ from AgNPs internalized by TNBC cells plays a role in the observed difference in the high sensitivity of TNBC cells to AgNPs compared to non‐malignant breast cells. The released Ag^+^ then causes DNA damage and ER stress, initiating the UPR. The UPR can repress DNA damage repair responses and sensitize cells to DNA damage.^68^ Therefore, the pleotropic stresses caused by AgNPs may be self‐reinforcing in TNBC and contribute to the sensitivity of TNBC cells to AgNPs. Induction of autophagy may also play a role in AgNP cytotoxicity,^76^ though we did not investigate this aspect in detail. However, we note that AgNPs and their degradation products can be found by TEM in autophagic vesicles in MDA‐MB‐231 cells (Figure 4) and SUM159 cells (Figure S7). Additionally, Torti et al found that the gene expression profile of TNBC cells under normal growth conditions was similar to non‐malignant breast cells under oxidative stress conditions, indicating that TNBC cells were already in a stressed state.^77^ Comparative studies by Bouwmeester and colleagues on gene expression following sub‐lethal AgNP exposure in Caco‐2 and MCF‐7 cells found that pathways connected to oxidative stress, responses to metal ions, or cell division were activated in both cell lines.^78^ Although Caco‐2 cells showed a higher sensitivity to AgNPs than MCF‐7 cells, no differences were observed between the two cell types related to which pathways were activated, but differences were observed in timing and magnitude, with responses being greater and more rapid in Caco‐2 cells as compared to MCF‐7 cells. The authors further noted that Caco‐2 cells appeared to have higher baseline stress levels than the MCF‐7 cells. Thus higher baseline stress levels in TNBC cells may limit their capacity to adapt to AgNP induced stress.

Intriguingly, the stroma of residual tumors on AgNP treated mice was highly infiltrated with inflammatory and plasma cells (Figure 8H). The reason for this remains unknown, and the nude mouse model required for human xenografts limits further assessment of AgNP induced immune responses in our study. A previous study in mice with fibrosarcoma showed increased leukocyte, lymphocyte, and granulocyte counts in mice after treatment with mouse serum albumin‐coated AgNPs at doses between 2 and 8 mg/kg.^79^ Likewise, intravenously dosed iron oxide nanoparticles reportedly induced immune cell infiltration in murine breast tumors.^12^ Stromal infiltration of plasma cells in breast and other cancers is correlated with increased overall survival.^80^ Additional research on the use of AgNPs for modulation of the tumor immune environment may lead to new opportunities to enhance immunotherapy of cancer. Furthermore, AgNPs can synergistically enhance chemotherapy,^18^ be formulated to co‐deliver drugs to cancer cells,^81^, ^82^ and act as sensitizers to ionizing radiation,^11^, ^19^ indicating that there are multiple potential clinical applications.

Although our data provide evidence that AgNPs can act as a safe and specific TNBC treatment, it is not possible to confirm that the PVP‐coated AgNPs we used for preclinical testing will exhibit a similar selectivity for TNBC and lack of toxicity in normal breast and other tissues in human cancer patients until human testing can be performed. There currently are no AgNP formulations approved clinically for intravenous delivery or for cancer therapy, but a recent case report from University of Texas Southwestern Medical Center describes a 78‐year old male who consumed AgNPs daily after failing all conventional chemotherapy, radiation, and surgery for nasal cavity squamous cell cancer metastatic to liver and lung. Within three months, all previously seen lung, liver, and lymph node metastases were no longer detectable by radiographic imaging, and complete resolution of the cancer has persisted for at least 36 months.^31^ No definitive conclusions regarding the clinical efficacy of AgNPs can be drawn from a single patient. However, the result suggests that AgNPs may exert anticancer effects in humans. This is supported by our experimental evidence, and we believe that further testing of AgNP‐based cancer therapy in animals and humans is justified.

## CONFLICT OF INTEREST

No authors have financial or other conflicts of interest related to the research described in this work.

## AUTHOR CONTRIBUTIONS

RS and JS designed the research, performed the experiments, and analyzed the data. CDF assisted with in vivo studies, cytotoxicity testing, performed western blots, and analyzed the data. BWB assisted with western blots, cytotoxicity studies, oxidative stress assays, and analyzed the data. IT and PAV performed 3D culture studies and analyzed the data. JJS performed studies with triangular silver nanoplates and AgNP degradation studies. AH assisted with nanoparticle characterization. GLD assisted with sample digestion and ICP‐MS analyses. KLC assisted with UPR studies and analyzed the data. WL performed histologic analysis on tumor sections. EA provided essential reagents and data analysis. CMF provided essential reagents and expertise for oxidative stress studies. RS, JS, CDF, and PAV wrote the manuscript. All authors critically reviewed and edited the manuscript.
