# Supplementary material for: Silver nanoparticles selectively treat triple‐negative breast cancer cells without affecting non‐malignant breast epithelial cells in vitro and in vivo
Source: FASEB Bioadv. 2019 Sep 30;1(10):639–60. doi: 10.1096/fba.2019-00021 (PMC6996381; doi:10.1096/fba.2019-00021)
Supplement: Supplementary file 1 [file FBA2-1-639-s001.pdf]

SUPPLEMENTARY FIGURES

Supplementary figure S1. Physicochemical characterization and cytotoxicity of PVP stabilized, triangular silver nanoplates.

Supplementary Figure S2. Physicochemical characterization and cytotoxicity of PVP stabilized, gold nanoparticles.

Supplementary Figure S3. Physicochemical characterization and cytotoxicity of chitosan-coated, triangular silver nanoplates.

Supplementary Figure S4. Physicochemical characterization and cytotoxicity of silica-shelled, triangular silver nanoplates.

Supplementary Figure S5. Evaluation of the effects storage and buffers on stability and cytotoxicity of AgNPs.

Supplementary Figure S6. TEM images of AgNPs in iMEC cells.

Supplementary Figure S7. TEM images of AgNPs in SUM159 cells.

Supplementary Figure S9. Cytotoxicity of AgNPs in S1 cell monolayer.

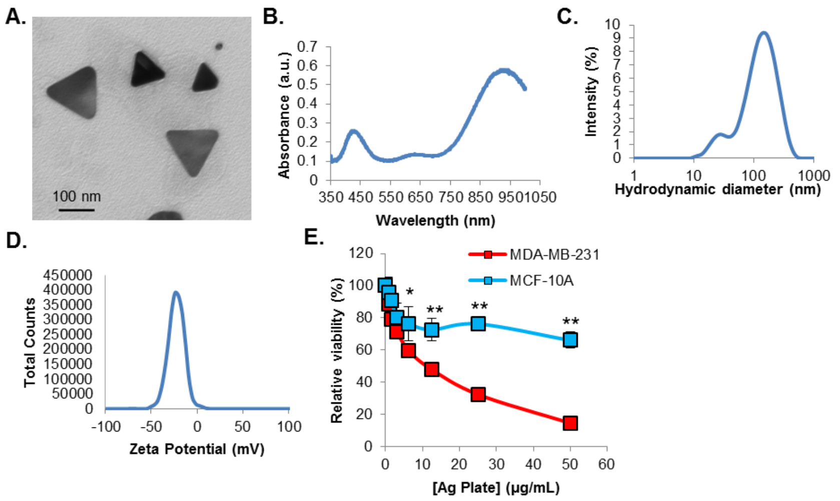

Supplementary Figure S1. *Physicochemical characterization and cytotoxicity of PVP stabilized triangular silver nanoplates.* (A) Transmission electron micrograph showing nanoparticle structure. (B) UV/vis spectroscopy showing plasmon resonance. (C) DLS evaluation of nanoparticle hydrodynamic diameter in water. (D) DLS evaluation of nanoparticle ζ-potential in water (pH 6.5). (E) Evaluation of nanoparticle cytotoxicity in MDA-MB-231 and MCF-10A cells after 48 h exposure. Cell viability was assessed by MTT assay. Data were obtained from 6 technical replicates per cell line and are representative of duplicate independent experiments. Statistical analysis was performed by two-way ANOVA and post-hoc Tukey Test. Significant differences are indicated (*p<0.5; **p<0.01).

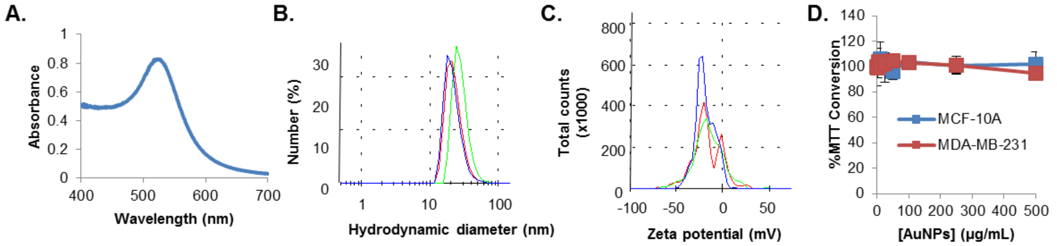

Supplementary Figure S2. *Physicochemical characterization and cytotoxicity of PVP stabilized, spherical gold nanoparticles (AuNPs).* (A) UV/vis spectroscopy showing plasmon resonance. (B) DLS evaluation of nanoparticle hydrodynamic diameter in water (shown in triplicate). (C) DLS evaluation of nanoparticle ζ-potential in water (pH 6.5; shown in triplicate). (D) Evaluation of nanoparticle cytotoxicity in MDA-MB-231 and MCF-10A cells after 48 h exposure. Cell viability was assessed by MTT assay. Data were obtained from 6 technical replicates per cell line and are representative of duplicate independent experiments. Statistical analysis was performed by two-way ANOVA and post-hoc Tukey Test. No significant differences were observed (p>0.5).

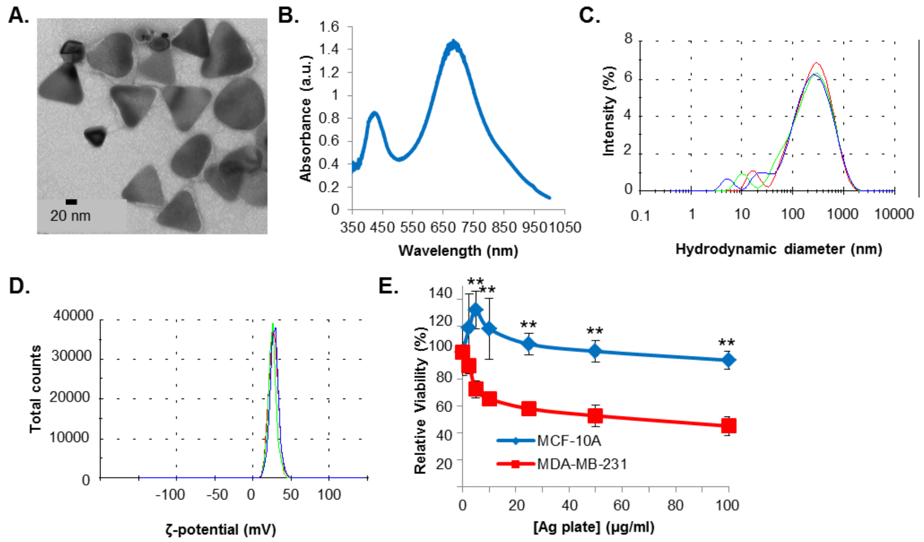

Supplementary Figure S3. *Physicochemical characterization and cytotoxicity of chitosan stabilized, triangular silver nanoplates.* (A) Transmission electron micrograph showing nanoparticle structure. (B) UV/vis spectroscopy showing plasmon resonance. (C) DLS evaluation of nanoparticle hydrodynamic diameter in water. (D) DLS evaluation of nanoparticle ζ-potential in water (pH 6.5). (E) Evaluation of nanoparticle cytotoxicity in MDA-MB-231 and MCF-10A cells after 48 h exposure. Cell viability was assessed by MTT assay. Data were obtained from 6 technical replicates per cell line and are representative of duplicate independent experiments. Statistical analysis was performed by two-way ANOVA and post-hoc Tukey Test. Significant differences are indicated **p<0.01).

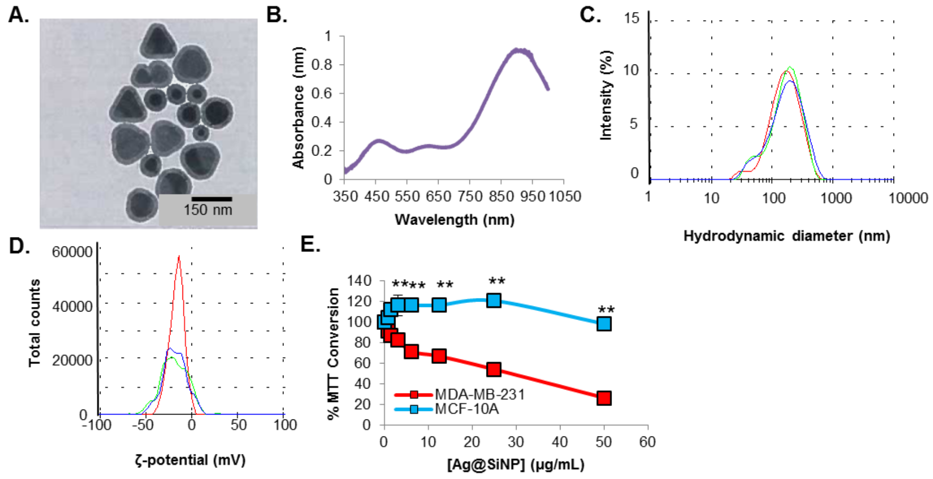

Supplementary Figure S4. *Physicochemical characterization and cytotoxicity of PVP stabilized, triangular silver nanoplates.* (A) Transmission electron micrograph showing nanoparticle structure. (B) UV/vis spectroscopy showing plasmon resonance. (C) DLS evaluation of nanoparticle hydrodynamic diameter in water. (D) DLS evaluation of nanoparticle ζ-potential in water (pH 6.5). (E) Evaluation of nanoparticle cytotoxicity in MDA-MB-231 and MCF-10A cells after 48 h exposure. Cell viability was assessed by MTT assay. Data were obtained from 6 technical replicates per cell line and are representative of duplicate independent experiments. Statistical analysis was performed by two-way ANOVA and post-hoc Tukey Test. Significant differences are indicated **p<0.01).

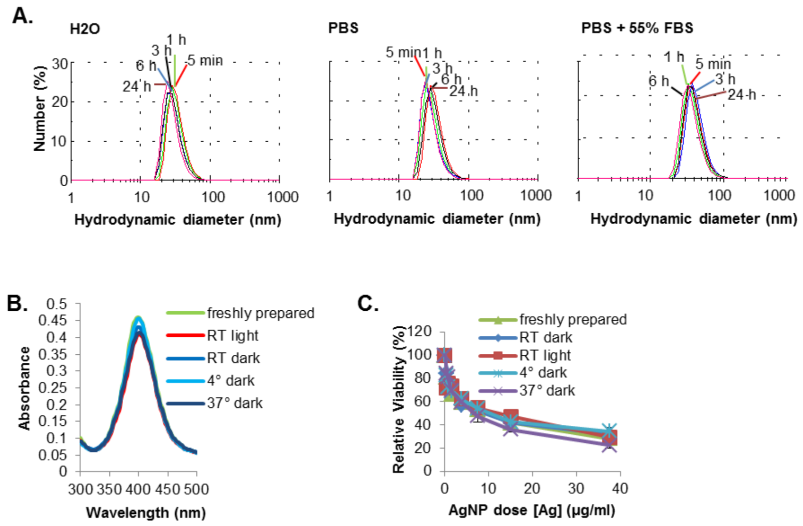

Supplementary Figure S5. *Evaluation of the effects storage and buffers on stability and cytotoxicity of AgNPs.* (A) The hydrodynamic diameter of PVP stabilized, 25 nm AgNPs was measured over time (5 min, 1 h, 3 h, 6 h, 24 h) in water, PBS, and PBS supplemented with 55% FBS. (B) The plasmon resonance of 25 nm AgNPs in PBS was evaluated for freshly hydrated particles, or after storage at 4 °C, room temperature (RT), or 37 °C in the dark or light for 1 week. (C) MDA-MB-231 cells were exposed to 25 nm AgNPs stored as in (B) and cell viability after 48 h exposure was assessed by MTT assay. Data were obtained from 6 technical replicates and are representative of duplicate independent experiments. Statistical analysis was performed by two-way ANOVA and no significant differences ($p>0.05$) were detected.

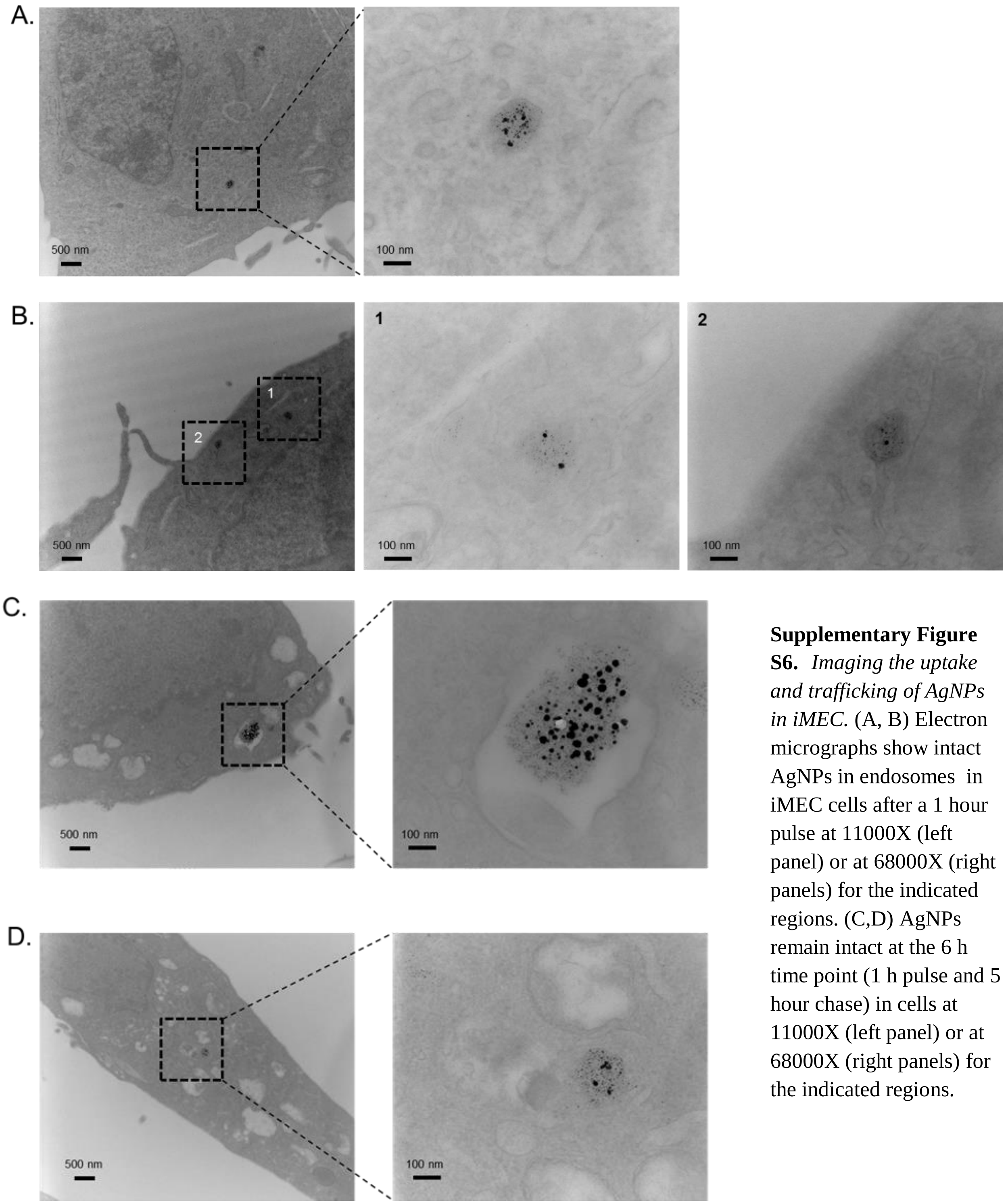

Supplementary Figure S6. *Imaging the uptake and trafficking of AgNPs in iMEC.* (A, B) Electron micrographs show intact AgNPs in endosomes in iMEC cells after a 1 hour pulse at 11000X (left panel) or at 68000X (right panels) for the indicated regions. (C,D) AgNPs remain intact at the 6 h time point (1 h pulse and 5 hour chase) in cells at 11000X (left panel) or at 68000X (right panels) for the indicated regions.

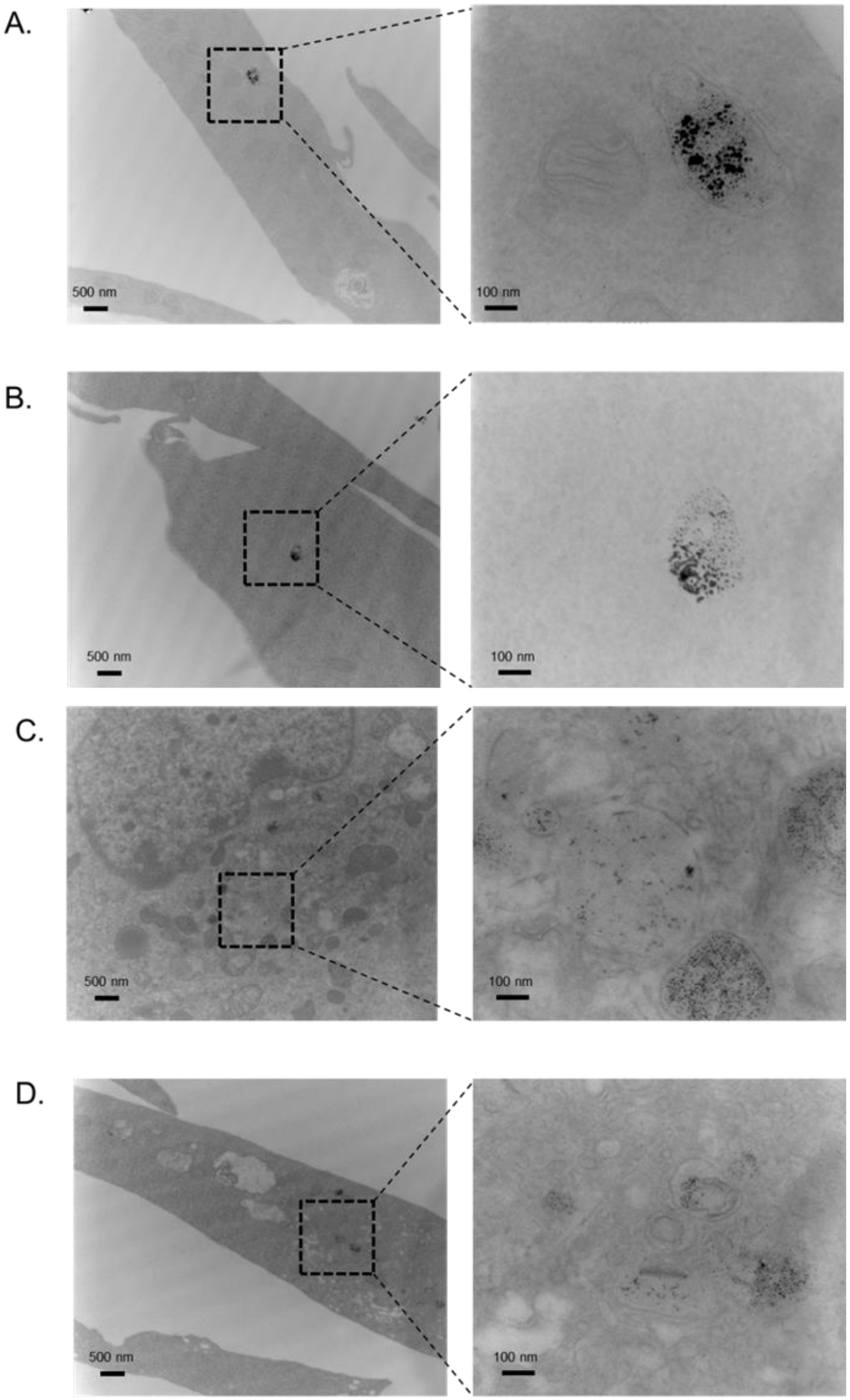

Supplementary Figure S7 *Imaging the uptake and trafficking of AgNPs in SUM159 cells* (A. B) Electron micrographs show AgNPs beginning to degrade in endosomes in SUM159 cells after a 1 hour pulse at 11000X (left panel) or at 68000X (right panels) for the indicated regions. (C,D) AgNPs are highly degraded at the 6 h time point (1 h pulse and 5 hour chase) in cells at 11000X (left panel) or at 68000X (right panels) for the indicated regions.

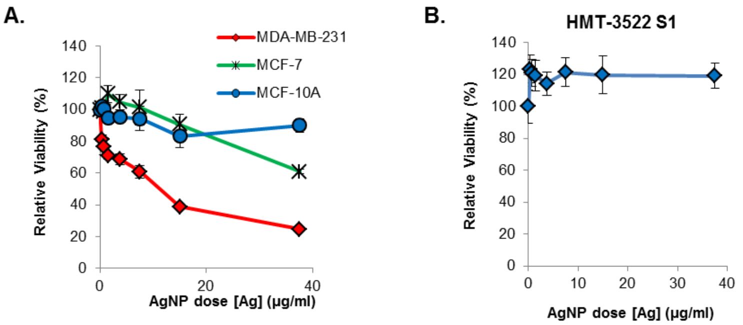

Supplementary Figure S8. *Cytotoxicity of AgNPs in cell monolayers.* (A) MDA-MB-231, MCF-7, and MCF-10A cells, or (B) HMT-3522 S1 normal mammary epithelial cells were exposed to PVP stabilized, 25 nm AgNPs for 48 h and viability was assessed by MTT assay. Data were obtained from 6 technical replicates per cell line and are representative of duplicate independent experiments.
